# Supplementary material for: Origin and recent expansion of an endogenous gammaretroviral lineage in domestic and wild canids
Source: Retrovirology. 2019 Mar 7;16:6. doi: 10.1186/s12977-019-0468-z (PMC6407205; doi:10.1186/s12977-019-0468-z)
Supplement: Supplementary file 2 — Additional file 2: Figure S1. Assembled CfERV breakpoints remapped to the CanFam3.1 reference. Three-way alignments for 58 non-reference insertions are shown. Alignments were used to depict CfERV-Fc1(a) LTR junctions obtained by assembled supporting reads (shown in red text) remapped to the CanFam3.1 reference sequence (shown in black text and underlined). The 5 bp sequence corresponding to the target site duplication is underlined and bolded in the reference allele. The coordinates of the CanFam3.1 reference sequence shown is provided above each alignment; the first base of the LTR is labeled and indicated by an asterisk shown respective of orientation (‘+’ or ‘−’). Insertions for which a provirus was validated are labeled as appropriate. The single assembled junctions are provided for either of two insertions: chr13:20,998,612 (3′ junction); chr27:44,066,943 (5′ junction). [file 12977_2019_468_MOESM2_ESM.pdf]

Figure S1

chr1:14872460-14872610 * chr1:14,872,531 (-)

TAGTGGGGATCCTGCTTCTCCCTCTCCTATGGGTACTCTCTCTTTCTCTCTTGCTCTCAAATGAA**TACAT**AAAATCTAAAAAAAAAAAAAAAAAGAAAAGAAAAGCTTCTTCAAAGGATTTTTATTTTTATTATGAATATTCCATAAACAG

TAGTGGGGATCCTGCTTCTCCCTCTCCTATGGGTGCTCTCTCTTTCTCTCTTGCTCTCAAATGAATACATTGTTAAGTTTTTTTAGTAACTAGACGGAAATGAGACCGCCAAGGCAAGCGAGGGTCCAAGAACAGATTT

ACTTCAGCCATTTTGGCGTATCCGCCATCTTGAGGAGTTTCCCTTCCCGCCTGGCCCCAACATACATAAAATCTAAAAAAAAAAAAAAAAAGTAAAGAAAAGCTTCTTCAAAGGATTTTTATTTTTATTATGAATATTCC

chr2:32862952-32863102

Additional 3’ sequence present in the LTR is underlined * chr2:32,863,024 (+)

CTTCCTGAGGGACCAGAGTCTCCATCAGCTTACAAACATCTTCCTGATTTACAAGCAGACAGCTTA**CTTAC**CAATAATCTAACTTCCAGATACCCATGGACTCAATTTCCTGGAGCCCTACCATCACCCTCCCCCCTCAGTAGAGATATT

TCCTGAGGGACCAGAGTCTCCATCAGCTTACAAACATCTTCCTGATTTACAAGCAGACAGCTTACTTACTGTCGGGGCCAGGCGGGAAGGGAAACTCCTCAAGATGGCGGATACGCCAAAATGGCTGAAG

ACCCTCGCTTGCCTTGGCGGTCTCATTTCCGTCTAGTTACTAAAAAAACTTAACATTTTAACACTTACCAATAATCTAACTTCCAGATACCCATGGACTCAATTTCCTGGAGCCCTACCATCACCCTCCCCCTCAGTAGAGATATT

chr2:36108257-36108407 * chr2:36,108,324 (-)

CTTAGACTCATTTCTGGCTGACAGGGTCCTGGTTCAAACATGCATTGAAGTCTTTTGCTTT**CTTTC**CTAGGGCTTTTCTGATGTCTTATCCCAGGACACTGGTGCTTGAAGGTACAGGTATCCCTAGAAGTTTGGGACTTACCACCTCTG

CTTAGACTCATTTCTGGCTGACAGGGTCCTGGTTCAAACATGCATTGAAGTCTTTTGCTTTCTTTCTGTTAAGTTTTTTTAGTAACTAGACGGAAATGAGACCGCCAAGGCAAGCGAGGGTCCAGGAACAGATTTTATTGCAGGCACCCT

CTTCAGCCATTTTGGCGTATCCGCCATCTTGAGGAGTTTCCCTTCCCGCCTGGCCCCAACACTTTCCTAGGGCTTTTCTGATGTCTTATCCCAGGACACTGGTGCTTGAAGGTACAGGTATCCCTAGAAGTTTGGGACTTACCACCTCTG

chr3:56080380-56080530 * chr3:56,080,478 (-)

CAGCCTTTCCCTCTGTTGGGTGGATTTTCTATGGAGTTTTGTCTGCATTGCCTTTATCCTCTCCTGCAGTGCTTTCAAACTTCGGTGTGCAC**AGAATCCCC**TGGACAACTGTTAAAATACAGATGCCCAGCCTCATCAGAGGTCTGCACC

CAGCCTTTCCCTCTGTTGGGTGGATTTTCTATGGAGTTTTGTCTGCATTGCCTTTATCCTCTCCTGCAGTGCTTTCAAACTTCGGTGTGCACAGAATTGTTAAGTTTTTTTAGTAACTAGACGGAAATGAGACCGCCAAGGCAA

CCCAAGTGGAGGTGGTGACAGGAACTTCAGCCATTTTGGCGTATCCGCCATCTTGAGGAGTTTCCCTTCCCGCCTGGCCCCAACAAGAATCCCCTGGACAACTGTTAAAATACAGATGCCCAGCCTCATCAGAGGTCTGCAC

chr4:22610476-22610626 Provirus * chr4:22,610,555 (+)

TTGCCTTGCCACTGACTCTTCCCCCTTGGATTGCAAGTATACCCAAGGCTCTTCCATCCTAAAAGAAGTCTTT**CCTCA**CTCATGATTTTCACTTTAGCTGTGGACTTTTTCTCACCTCCCCTTCACTACTCTTTGAAACAATCACCTACA

TTGCCTTGCCACTGAGTCTTCCCCCTTGGATTGCAAGTATACCCAAGGCTCTTCCATCCTAAAATAAGTCTTTCCTCATGTTGGGGCCAGGCGGGAAGGGAAACTCCTCAAGATGGCGGATACGCCAAAATGGCTGAAGTTCCTGTCACC

CTGTTCTTGGACCCTCGCTTGCCTTGGCGGTCTCATTTCCGTCTAGTTACTAAAAAAACTTAACACCTCACTCATGATTTTCACTTTAGCTGTGGACTTTTTCTCACCTCCCCTTCACTACTCTTTGA

chr5:10128684-10128834 Provirus * chr5:10,128,780 (-)

AGGCTTTGGATATATGGTATACCACTTTCCTTTTCCAGGTAGTAGCTTGCAACTGGGGAAATTTGTCCCAATTACATGTGGTATGTCAACA**TAGGT**ATTATAACAAGAGGGTATCTTGAATTTCCCTAACAGCTTCAATATGTCTGGTTT

TCATCCCAAGTGGAGGTGGTGACAGGAACTTCAGCCATTTTGGCGTATCCGCCATCTTGAGGAGTTTCCCTTCCCGCCTGGCCCCAACAATAGGTATTATAACAAGAGGGTATCTTGAATTTCCCTAACAGCTTCAATATGTCTGGT

GGCTTTGGATATATGGTATACCACTTTCCTTTTCCAGGTAGTAGCTTGCAACTGGGGAAATTTGTCCCAATTACATGTGGTATGTCAACATAGGTGTTAAGTTTTTTAGTAACTAGACGGAAATGAGACCGCCAAGGCAAGCGAGGGTC

chr5:16865413-16865563 * chr5:16,865,484 (-)

CTGCAGGCTGCTTTAGGTGAGGGCCAGGAAGTTCAAATGACAGAGGTTTCCCCAGTCTGATTTCA**GAATC**TAGCACTGATAGGGCTCTGGTTGGGGAGCCCCAGGCCAGGTGGTGCCCATCCCATGACAACTCAAGAGAAAGTGTCTGTT

GCAGGCTGCTTTAGGTGAGGGCCAGGAAGTTCAAATGACAGAGGTTTCCCCAGTCTGATTTTAGAATCTGTTAAGTTTTTTTAGTAACTAGACGGAAATGAGACCGCCAAGGCAAGCGAGGGTCCAAGAACAGATTTTATTG

GGAACTTCAGCCATTTTGGCGTATCCGCCATCTTGAGGAGTTTCCCTTCCCGCCTGGCCCCAACAGAATCTAGCACTGATAGGGCTCTGGTTGGGGAGCCCCAGGCCAGGTGGTGCCCATCCCATGACAACTCAAGAGAAAGTGTCTG

chr5:78331508-78331658 Provirus * chr5: 78,331,579 (-)

GAACAACAGTAACTTACGTTTCCGTGGTGCTTTAACCGCGCTTCACTTGCGCTCTCCACGTGACC**TTGAC**TACATCCCACTTGGGAAGATGAGGACAGCAGCATCCTCGATAAGGAAGGGTCTGGATTCAGTGTGTGAGGCGCGCGGGCC

AGTAACTTACATTTCCGTGGTGCTTTAACCGCGCTTCACTTGCGCTCTCCACGTGACCTTGACTGTTAAGTTTTTTTAGTCCAAGACAAGGTGGTAGGGGGTTTACATAACGTTGTAAGGCAGAACGGTTTCCTATTGGT

GAACTTCAGCCATTTTGGCGTATCCGCCATCTTGAGGAGTTTCCCTTCCCGCCTGGCCCCAACATTGACTACATCCCACTTGGGAAGATGAGGACAGCAGCATCCTCGATAAGGAAGGGTCTGGATTCAGTGTGTGAGGCGCGCGGGCC

chr5:80814502-80814652 (5’ bases 1-141 present only) * chr5:80,814,713 (+)

AGAAAGAAAGAAAGGTTCTTGATAATGGTCTTCATTATTTCACACTTATACTGAGGACAATTTGCTGAGAACAAGGTACAAAATATAAGGAGAAACCAGATCCAACTTTAACAGGGAAATTCCTCATGAGAGAAAAATAAATCTTTAAAA

AGAAAGAAAGAAAGGTTCTTGATAATGGTCTTCATTATTTCACACTTATACTGAGGACAATTTGCTGAGAACAAGGTGTTGGGGCCAGGCGGGAAGGGAAACTCCTCAAGATGGCGGATACGCCAAAATGGCTGAAGTTCCTGTCACCA

AATGGCTGAAGTTCCTGTCACCACCTCCACTTGGGATGACAGCTTGAGCAGACCCTTACACCTCTCCTTTGGACTTCCTCAACCGAACCCAGATCCAACTTTAACAGGGAAATTCCTCATGAGAGAAAAATAAATCTTTA

chr6:52819059-52819209 * chr6:52,819,151 (+)

CTACTATGGAGATAAGGCAAAAAAAAAAAATGCAAACAATATGGCTTTTCCAGAAAAAAGAAGTACACTTAAATGTATACTACAGTG**TTAAT**GAATGATGTCAGATCTTTTTCTGAAGTGCAGATTTTGATGGAAGAAAAAGGGAGCAAAG

AAAAAAATGCAAACAATATGGCTTTTCCGGAAAAAAGAAGTACACTTAAATGTATACTACAGTGTTAATTGTTGGGGCCAGGCGGGAAGGGAAACTCCTCAAGATGGCGGATACGCCAAAATGGCTGA

AAAATCTGTTCTTGGACCCTCGCTTGCCTTGGCGGTCTCATTTCCGTCTAGTTACTAAAAAAAAAAAAACTTAACATTAATGAATGATGTCAGATCTTTTTCTGAAGTGCAGATTTTGATGGAAGAAAAAGGGAGCA

chr7:6132651-6132801 * chr7:6,132,731 (+)

GGAAGCATAGGATTGGTGATCAGGGTAGGACAGCATGCTGTCTCTGGTATTGTGATTTATTATAAAGCTGGAGTA**ATCAC**AAATGTCATATTCCGAGAGGATAGACAAACAGATCAGTGGAGAAGAATAAGAGATGCCAGAAACCATCCC

GCATAGGATTGGTGATCAGGGTAGGACAGCATGCTGTCTCTGGTATTGTGATTTATTATAAAGCTGGAGTAATCACTGTTGGGGCCAGGCGGGAAGGGAAACTCCTCAAGATGGCGGATACGCCAAAATGGCTGAAGTTCCTGTCA

ATAAAATCTGTTCTTGGACCCTCGCTTGCCTTGGCGGTCTCATTTCCGTCTAGTTACTAAAAAAAACTTAACAATCACAAATGTCATATTCCGAGAGGATAGACAAACAGATCAGTGGAGAAGAATAAGAGA

chr8:12837599-12837749 * chr8:12,837,689 (+)

TGTCAGACAAGATTAGGTCTCTTGTGGAGGCTCGACTGGGGAAGTACATGCTTCCTCGTGCTCATGGTTGTTGGAAGTATTCTG**GTCAT**TACAGCCATAGGATCCATGGCAACTTACTTACTCAAAGCCAGTGACAGAAAGAGAGAGGTT

CGACTGGGGAAGTACATGCTTCCTCGTGCTCATGGTTGTTGGAAGTATTCTGGTCATTGTTGGGGCCAGGCGGGAAGGGAAACTCCTCAAGATGGCGGATACGCCAAAATGGCTGAAG

GCCTGCAATAAAATCTGTTCTTGGACCCTCGCTTGCCTTGGCGGTCTCATTTCCGTCTAGTTACTAAAAAACTTAACAGTCATTACAGCCATAGGATCCATGGCAACTTACTTACTCAAAGCCAGTGACAGAAAGAGAGAGGTT

chr8:18375890-18376040 * chr8:18,375,962 (+)

TGCCCCTATTTTGTTTATTGTTGACCACATAGAATTAAACTAGAAATTTAATCCAAAAGGGAACTC**AAAAT**GTTCATATTTCTGGATATTGAAATATATTATATAATAACCCATATGTGAAAGATGAAGTTGTGATTGAAATGGGAGTTT

GCCCTTATTTTGTTTATTGTTGGCCACATAGAATTAAACTAGAAATTTAATCTAAAAGGGAACTCAAAATTGTTGGGGCCAGGCGGGAAGGGAAACTCCTCAAGATGGCGGATACGCCAAAATGGCTGAGGTTCCTGTCACCACCTCC

ATCTGTTCTTGGACCCTCGCTTGCCTTGGCGGTCTCATTTCCATCTAGTTACTAAAAAACTTAACAAAAATGTCCATATTTCTGGATATTGAAATATATTATATAATAACCCATATGTGAAAGATGAAGTTGTGATTGAAATGGGAGATT

chr9:9097690-9097840 * chr9:9,097,758 (+)

ATGAGTTATCTGAATCCATCAATCTATTAAATACTGAATGAGCTGTAAGCCTTTCTTAAATA**ATAGT**TAAGTCTTTAcctaactctgggaaatgaacaaggggtagtggaaggggaggtgggcaggggagttggggtgactgggtgatgg

GAGTTATCTGAATCCATCAATCTATTAAATACTGAATGAGCTGTAAGCCTTTCTTAAATAATAGTTGTTGGGGCCAGGCGGGAAGGGAAACTCAAGATGGCGGATACGCCAAAATGGCTGAAGTTCCTGTCACCACCTCCACTT

TGGACCCTCGCTTGCCTTGGCGGTCTCATTTCCGTCTAGTTACTAAAAAACTTAACAATAGTTAAGTCTTTACCTAACTCTGGGAAATGAACAAGGGGTAGTGGAAGGGGAGGTGGGCAGGGGAGTTGGGGTGAC

chr9:15385643-15385793 * chr9:15,385,714 (-)

GTAGGTACTATTATTGTCACCACATATGCAACTGGGAAGAGCTGAAATGGGGATTTGAGCCTGGG**GACCC**GACTTCTGCCAATGTAAAACGTAATATGATTTCCTTGCTGTGTGATTTCAGACTTTTTGTCACCTCTATTCTGACAAATT

TAGGTACTATTATTGTCACCACATATGCAACTGGGAAGAGCTGAAATGGGGATTTGAGCCTGGGGACCCTGTTAAGTTTTTTAGTAACTAGACGGAAATGAGACTGCCAAGGCAAGCGAGGGTCCAAGAACAGATTTTATTGCAGG

CTCAGCCATTTTGGCGTATCCGCCATCTTGAGGAGTTTCCCTTCCCGCCTGGCCCCAACAGACCCGACTTCTGCCAATGTAAAACGTAATATGATTTCTTTGCTGTGTGATTTCAGACTTTTTGTCACCTCTAT

chr9:29215716-29215866 * chr9:29,215,798 (+)

CAAAATAAAACCTAACAAGATTGATAATGTTTTCTCCCATTCTATTTGAGATAGATCTTTTATATACTATTATGTT**TTAAT**GAAGTAGGAAACAAGGCCCATCTCAAAGGTCCCATTCACAATCTAGAAATATTCTAGAACAACTTTCTG

AAAATAAAACCTAACAAGATTGATAATGTTTTCTCCCATTCTATTTGAGATAGATCTTTTATATACTATTATGTTTTAATTGTTGGGGCCAGGCGGGAAGGGAAACTCCTCAAGATGGCGGATACGCCAAAATGGCTGAAGTTCCT

TGCAATAAAATCTGTTCTTGGACCCTCGCTTGCCTTGGCGGTCTCATTTCCGTCTAGTTACTAAAAAAACTTAACATTAATGAAGTAGGAAACAAGGCCCATCTCAAAGGTCCCATTCACAATGTAGAAATATTCTAGAAC

chr10: 67519279-67519434 * chr10:67,519,359 (-)

AGTGGAAGCAAGAAACTAGTTAGTAAACATTGGAATTGACTGAAAAAAGACGATGGCATGGGGACCAGAGAATGG**AGTAG**ATCTAGTGAGAAGTGGCAGATAGATTTTGATGGCAGAGCCAATAGGATCTCTGGACATCCTGGATATGGAG

AAGCAAGAAACTAGTTAGTAAACATTGGAATTGACTGAAAAAAGACGATGGCATGGGGACCAGAGAATGGAGTAGTGTTAAGTTTTTTAGTAACTAGACGGAAATGAGACCGCCAAGGCAAGCGAGGGTCCAAGAACAGATTTT

GGTGGTGACAGGAACTTCAGCCATTTTGGCGTATCCGCCATCTTGAGGAGTTTCCCTTCCCGCCTGGCCCCAACAAGTAGATCTAGTGAGAAGTGGCAGATAGATTTTGATGGCAGAGCCAATAGGATCTCTGGACATCCTGG

chr11:7046350-7046500 * chr11:7,046,441 (-)

TAATTTTTTGAGGAACCTCCACACTGTTTTCTGCATTAACTACATCAATTTACATTTCTACCAGTAGGGCACAAGGATAGGGATC**CCT**TCCTTCACCTTAAGATTTGGTGTCGAGGAGGACAGTATTAAAAATTTTATTATACCTGGGAC

TTTGAGGAACCTCCACACTGTTTTCTGCATTAACTACATCAATTTACATTTCTACCAGTAGGGCACAAGGATAGGGATCCCTTCTGTTAAGTTTTTTAGTAACTAGATGGAAATGAGACCGCCAAGGCAAGCGAGGGTCCAA

AAGTGGAGGTGGTGACAGGAACTTCAGCCATTTTGGCGTATCCGCCATCTTGAGGAGTTTCCCTTCCCGCCTGGCCCCAACACCTTCCTTCACCTTAAGATTTGGTGTCGAGGAGGACAGTATTAAAAATTTTATTATACCTGGG

chr11:6426768-6426918 * chr11:6,426,854 (+)

AGGCTTGGTTCGTGTCAGTGCAAATAACATGGAGGGAAGACCGGAGGGTTCTTTCTACTGACTGTTTCTTTTTTTAGGTA**GTAGT**GATGACTATTGATTGGATACCATTTATTTATTTTATTGCCACTTTTTAGGGAGTGTTTACCATCT

GCTTGGTTCGTGTCAGTGCAAATAACACGGAGGGAAGACCGGAGGGTTCTTTCTACTGACTGTTTCTTTTTTTAGGTAGTAGTTGTTGGGGCCAGGCGGGAAGGGAAACTCCTCAAGATGGCAGATACGCCAAAATG

CCTGCAATAAAATCTGTTCTTGGACCCTCGCTTGCCTTGGCGGTCTCATTTCCGTCTAGTTACTAAAAAAAACTTAACAGTAGTGATGACTATTGATTGGATACCATTTATTTATTTTATTGCCACTTTTTAGGGAGTGTTTAC

chr11:48336263-48336413 * chr11:48,366,348 (-)

GCTAAACCGCTGAGCCACCCAGGCTGCTCTCTCTGTCTCTCTCAAATGAATAAATTTTTTTTTGAATCTTTTTTTAAAG**AAGAT**AAGATAGTACTGCACCTCCTGCTCACAAAGTACTGGAAATGGCGATTGTTTCCTCCAGTCAGAGTA

TAAACCGCTGAGCCACCCAGGCTGCTCTCTCTGTCTCTCTCAAATGAATAAATTTTTTTTTGAATCTTTTTTTAAAGAAGATTGTTAAGTTTTTTAGTAACTAGACGGAAATGAGACCGCCAAGGCAAGCGAGGG

TGGAGGTGGTGACAGGAACTTCAGCCATTTTGGCGTATCCGCCATCTTGAGGAGTTTCCCTTCCCGCCTGGCCCCAACAAAGATAAGATAGTACTGCACCTCCTGCTCACAAAGTACTGGAAATGGCGATTGTTTCCTCCAGTCAGAG

chr12:869800-869950 Provirus * chr12:869,873 (-)

CACCAAGTCTGATCAAATTACATATGAGGATTTTGAGACGCCAGTGTGCTCTTGATTTTCAGTGCTT**GGGAG**GTTACAAGAAACACCAGGAGGTAAACTTATCATGGAAAATAGAGCCCAGGTGTGTGTGCACAGGCACCTGTGTCTACT

GTCTGATCAAATTACATATGAGGATTTTGAGACGCCAGTGTGCTCTTGATTTTCAGTGCTTGGGAGTGTTAAGTTTTTTTTAGTAACTAGACGGAAATGAGACCACCAAGGCAAGCG

CAGGAACTTCAGCCATTTTGGCGTATCCGCCATCTTGAGGAGTTTCCCTTCCCGCCTGGCCCCAACAGGGAGGTTACAAGAAACACCAGGAGGTAAACTTATCATGGAAAACAGAGCCCAGGTGTGTGTGCA

chr12:9415607-9415757 * chr12:9,415,667 (-)

ATTAAAAGTAGAATTTTAAGAATTTTTTTTTATTGCTATGTAGAAATCTAGTTG**CTTTT**AGTATTTGACCTTACTTCTTGTTATCTTTCTAAAGTCATTTATTATTCTAGTAATTGTTTTGTAGTACAAATCCTGATTCCTGGGCATTTT

ATTAAAAGTAGAATTTTAAGAATTTTTTTTTATTGCTATGTAGAAATCTAGTTGCTTTTTGTTAAGTTTTTTAGTAACTAGACGGAAATGAGACCGCCAAGGCAAGCGAGGGTCCAAGAACAGATTTTATTGCAGGCA

CATTTTGGCGTATCCGCCATCTTGAGGAGTTTCCCTTCCCGCCTGGCCCCAACACTTTTAGTATTTGACCTTACTTCTTGTTATCTTTCTAAAGTCATTTATTATTCTAGTAATTGTTTTGTAGTACAAATCCTGATTCCTGGGCATTTT

chr13:10866571-10866721 * chr13:10,866,647 (+)

ACATTCCAAACATAATCTTCTTTTTACTATTATAAAGTAGAAACCTAATTTCCTTAGAGTACAGTAGCTC**TTAAC**CTAAATATCTCACCTGGGAGAGTAAGATTCTTTAATTTTTTATTCACTATCATTAGGAACCTAAAGTGGTCTGGT

TAATTCCAAACATAATCTTCTTTTTACTATTATAAAGTAGAAACCTAATTTCCTTAGAGTACAGTAGCTCTTAACTGTTGGGGCCAGGCGGGAAGGGAAACTCCTCAAGATGGCGGATACGCCAAAATGGCTGAAGTTCC

AAATCTGTTCTTGGACCCTCGCTTGCCTTGGCGGTCTCATTTCCGTCTAGTTACTAAAAAAAACTTAACATTAACCTAAATATCTCACCTGGGAGAGTAAGATTCTTTAATTTTTTATTCACTATCATTAGGAACCTAAAGTGGTCTGGT

chr13:16157716-16157866 * chr13:16,157,778 (+)

CTTACTACCAATACTCTGGTCTTCCTCTAATTTCATATTTTTCCTTAAAACAGATA**TGAAC**ATTATCATAGTATTCACAAAGAGCCTTCATAGTTGCTTACTGTCAATGGCATAAAGGCCAAACTGTTTAACAAAATGGtcacaacagac

CTTACTACCAATACTCTGGTCTTCCTCTAATTTCATATTTTTCCTTAAAACAGATATGAACTGTTGGGGCCAGGCGGGAAGGGAAACTCTTCAAGATGGCGGATACGCCAAAATGGCTGAGGTTCCTGTCACCACC

CCCTCGCTTGCCTTGGTGGTCTCATTTCCGTCTAGTTACTAAAAAAAACTTAACATGAACATTATCATAGTATTCACAAAGAGCCTTCATAGTTGCTTACTGTCAATGGCATAAAGGCCAAACTGTTTAACAA

chr13:20998526-20998676 * chr13: 20,998,612 (-)

ATACTAAAACCAATTTTTACAAAAAAAAAATTTACTATGACCATGCACAATTCTAAACAATGTCAATGATAATACACTCTCCCTACCAGGACAGACCACTCCCACCATCACTTCTCCCCCTTAATAGCCACTCACTGCCTGTCCCAAAGG

TACTAAAACCAATTTTTACAAAAAAAAAATTTACTATGACCATGCACAATTCTAAACAATGTCAATGATAATACACTCTCCCTACTGTTAAGTTTTTTAGTAACTAGACGGAAATGAGACCGCCAAGGCAAGCGAGGGTCCAAGAAC

chr13:32380467-32380617 Provirus * chr13:32,380,539 (+)

TTTGCATTTTGTCCTGGAACCCACCCCATTCATCTCCTTTGTTATATTCTTTCTACAGGTGATTTC**ATCAG**CCTCCTAGATTTTATCAATGTCTATGCATTAATGATTCCAAACTATTCGTTTAAGCTTCTTACTCTTCCTGAGCTCCAA

TTGCATTTTGTCCTGGAACCCACCCCATGCATCTCCTTTGTTATATTCTTTCTACAGGTGATTTCATCAGTGTTGGGGCCAGGCGGGAAGGGAAACTCCTCAAGATGGCGGATACGCCAAAATGGCTGAAGTTCCTGTCACCACC

TCTGTTCTTGGACCCTCGCTTGCCTTGGCGGTCTCATTTCCGTCTAGTTACTAAAAAAACTTAACAATCAGCCTCCTAGATTTTATCAATGTCTATGCATTAATGATTCCAAACTATTCGTTTAAGCTTCTTACTCTTCCTGAGCTCCAA

chr13:17413370-17413520 * chr13:17,413,419 (-)

TGTATGGGATCCCTGGGTGGCGCAGCGGTTTGGCGCCTGCCTTTGGCCCAGGGCGCGATCCTGGAGACTC**AGGAT**CGAATCCCACATCGGGCTCCCGGTGCATGGAGCCTGCTTCTCCCTCTGCCTATGTCTCTGCCTCTCTCTCTCTCT

GGGATCCCTGGGTGGCGCAGCGGTTTGGCGCCTGCCTTTGGCCCAGGGCGCGATCCTGGAGACTCGGGATTGTTAAGTTTTTTAGTAACTAGACGGAAATGAGACCGCCAAGGCAAGCGAGGGTCCAAGAACAGATTTTATTGC

TGACAGGAACTTCAGCCATTTTGGCGTATCCGCCATCTTGAGGAGTTTCCCTTCCCGCCTGGCCCCAACAGGGATCGAATCCCACATCGGGCTCCCGGTGCATGGAGCCTGCTTCTCCCTCTGCCT

chr15:32084913-32085063 * chr15:32,084,977 (-)

ATTAAACTAAGTAAGAGAAATGAGTTGAAAAATGTTACATACCCTATGTTTCCATGTA**TATGT**TATTTTGGAAAAAGAAGAAAAATTGTAACAACAAAAATAAAAATAAATGGTTTGCAGGGGCTGAGGGTAGATGGAATCTCCCTTATT

ATTAAACTAAGTAAGAGAAATGAGTTGAAAAATGTTACATACCCTATGTTTCCATGTATATGTTGTTAAGTTTTTTAGTAACTAGACGGAAATGAGACCGCCAAGGCAAGCGAGGGTCCAAGAACAGATTTTATTGCAGGCACC

TGAGGAGTTTCCCTTCCCGCCTGGCCCCAACATATGTTATTTTGGAAAAAGAAGAAAAATTGTAACAACAAAAATAAAAATAAATGGTTTGCAGGGGCTGAGGGTAGATGGAAT

chr16:6873721-6873871 * chr16:6,873,790 (-)

AATGGGAAGGGCTGAGTGGGTCTGTAGAGACAATTACACCCTCTGGGTAACCATTTTTATGAA**GGTGA**GAGAAAGCACTGACCACTAACGGAAGAGAACCAAGGCTACGTGACAGCACAGTTTTGAGTTTCCTTAATTACCTGAAAGCCC

TGGGAAGGGCTGAGTGGGTCTGTAGAGACAATTACACCCACTGGGTAACCATTTTTATGAAGGTGATGTTAAGTTTTTTAGTAACTAGACGGAAATGAGACCGCCAAGGCAAGCGAGGGTCCAAGAACAGATTTTATTGCAGGCACC

CCTCAGCCATTTTGGCGTATCCGCCATCTTGAGGAGTTTCCCTTCCCGCCTGGCCCCAACAGGTGAGAGAAAGCACTGACCACTAACGGAAGAGAACCAAGGCTACGTGACAGCACAGTTTTGAGTTTCCTTAATTACCTGAAAGCCC

chr17:30368704-30368854 * chr17:30,368,796 (-)

CATGATTCAAAGGAAGAAATTTTCCTCTATATATTATCTACTGTTGATAAATCCTTGGTCAGTGAATCACCTCCTCTGATTTCTAT**CCATC**CTCTTCACTATTCTTGGGAGGATAGGTCACATCAGGGATACTTCTCCACAATCTCACTG

CATGATTCAAAGGAAGAAATTTTCCTCTATATATTATCTACTGTTGATAAATCCTTGGTCAGGGAATCCCCCCCTCTGATTTCTATCCATCTGTTAAGTTTTTTTAGTAACTAGACGGAAATGAGACCGCCAAGGCAAGCGAGGGTC

TGGCGTATCGGCCATCTGGAGGAGTTTCCCTTCCCGCCTGGCCCCAACACCATCCTCTTCCCTATTCTTGGGAGGATAGGTCACATCAGGGATACTTCTCCACAATCTCACTG

chr17:9744887-9745037 Provirus * chr17:9,744,973 (-)

TGTGCAATCTCCCAGAAAGCCTGATAAATACACAGCAGATGTAGCCACGCTTTAGAACTTGCAAGTCTTTTAAAAACATT**ATCTT**GTTTCATAGTAATCACTTCCCTTTTTAGGTACTTTGTAAATTATTTTCATTGTTCGTTTTTTTTT

TGTGCAATCTCCCAGAAAGCCTGATAAATACACAGCAGATGTAGCCACGCTTTAGAACTTGCAAGTCTTTTAAAAACATTATCTTTGTTAAGTTTTTTTAGTAACTAGACGGAAATGAGACCGCCAAGGCAAGCGAGGGTCCAAGAACAG

GTGGAGGTGGTGACAGGAACTTCAGCCATTTTGGCGTATCCGCCATCTTGAGGAGTTTCCCTTCCCGCCTGGCCCCAACAATCTTGTTTCATAGTAATCACTTCCCTTTTTAGGTACTTTGTAAATTATTTTCATTGTTCGTTTTTTTT

chr19:9813589-9813739 * chr19:9,813,667 (-)

GTGAAGCCATAAATTTAATAGAACTTAAAAATATTTAAGGTACATTACCATTTTGAGTTCCCATTCCATCTG**GTACT**ATGCTATACAAACAGCCGTGTGGAGAAATTAAACTTTCCTGGGTTTCTCAGTGCCTCTTGGGACTCTTCACTT

GAAGCCATAAATTTAATAGAACTTAAAAATATTTAAGGTACATTACCATTTTGAGTTCCCATTCCATCTGGTACTTGTTAAGTTTTTTTAGTAACTAGACGGAAATGAGACCGCCAAGGCAAGCGAGGGTCCAAGAACAGGCACCCT

GTGACAGGAAGTTCAGCCATTTTGGCGTATCCGCCATCTTGAGGAGTTTCCCTTCCCGCCTGGCCCCAACAGTACTATGCTATACAAACAGCAGTGTGGAGAAATTAAACTTTCCTGGGTTTCTCAGTGCCTCTTGGGACTCTTCACT

chr20:14974909-14975059 * chr20:14,974,979 (+)

ACATCTTTAAATAGGAACTATGTTGGTTTAAGTGAGATGACATAAATGGAAGTTTTCGCAAATG**AAAAA**TACAAAAACGGTTATCAGGTTTTGGTACATTACTTAGTGATTTTCAGAAACTGACCTAAATAGTTTTGGCGGTCAGATTAG

CTTTAAATAGGAACTATGTTGGTTTAAGTGAGATGACATAAATGGAAGTTTTCGCAAATGAAAAATGTTGGGGCCAGGCGGGAAGGGAAACTCCTCAAGATGGCGGATACGCCAAAATGGCTGAAGTTCCTGTCACCAC

TGTTCTTGGACCCTCGCTTGCCTTGGCGGTCTCATTTCCGTCTAGTTACTAAAAAAACTTAACAAAAAATACAAAAACGGTTATCAGGTTTTGGTACATTACTTAGTGATTTTCAGAAACTGACCTAAATAGTTTTGGCGGTCAG

chr20:12058377-12058527 * chr20:12,058,450 (+)

TAACAAGTAGGCTGTTAGTCTTGTTGGAGATCCCTTATCAGTAATGAGTTTTGATTTTTTTTAACTG**CTTTC**TAATTTTTTTCTTCTTTAATTTGGCTTTCAACGTTTGAGTATGATGCGTCTGGCCATCAATCTCTTTGCATTTATCCT

TAACAAGTAGGCTGTTAGTCTTGTTGGAGATCCCTTATCAGTAATGAGTTTTGATTTTTTTTAACTGCTTTCTGTTGGGGCCAGGCGGGAAGGGAAACTCCTCAAGATGGCGGATACGCCAAAATGGCTGAGGTTCTTGTCAC

ATCTGTTCTTGGACCCTCGCTTGCCTTGGCGGTCTCATTTCCGTCTAGTTACTAAAAAACTTAACACTTTCTAATTTTTTTCTTCTTTAATTTGGCTTTCAACGTTTGAGTATGATGCGTCTGGCAATCAATCTC

chr20:16677067-16677217 * chr20:16,677,142 (+)

GTTATATAGCAAAACAAAAAAAAAATGTTTATCTGTCTTTAGTAATTGGGAAGAGAGTCTTTCTAAACTA**AATA**TGTCACAGTGAATTGAGAAAATATAATACCAAAGTCAGAGAAACATCAGAAAAAACGCAAGTTGGAAAAATACACT

GTTATATAGCAAAACAAAAAAAAAATGTTTATCTGTCTTTAGTAATTGGGAAGAGAGTCTTTCTAAACTAAATAAGTTGGGGCCAGGCGGGAAGGGAAACTCCTCAAGATGGCGGATATGCCAAAATGGCTGAAGTTCCTGTCACCACCT

TCTGTTCTTGGACCCTCGCTTGCCTTGGGGTCTCATTTCCGTCTAGTTACTAAAAAAACTTAACAAATATGTCACAGTGAATTGAGAAAATATAATACCAAAGTCAGAGAAACATCAGAAAAAAAAGCAAGTTGGAAAATACAC

chr21:9814285-9814435 *chr21:9,814,350 (+)

TGTTCCATATGCTTTCTATAAATCTAAAACTCTTCTATAAGATGTTAATTTAAAAAATA**CCTAT**CAGGTATAATCTATAGGATTGCTGTAAAATTCAAGTGAGATAAATAAATAAATAAATAAATAAATAAATAAATAAATAAATAATTT

TGTTCCATATGCTTTCTATAAATCTAAAACTCTTCTATAAGATGTTAATTTAAAAAATACCTATTGTTGGGGCCAGGCGGGAAGGGAAACTCCTCAAGATGGCGGATACGCCAAAATGGCTGAAGTTCCTGTCACCA

TGGACCCTCGCTTGCCTTGGCGGTCTCATTTCCGTCTAGTTACTAAAAAAACTTAACACCTATCAGGTATAATCTATAGGATTGCTG

chr21:7250000-7250150 * chr21:7,250,064 (-)

TGCTCTACTCGTGGCCTTTGCTGAAGGTATCCTCCATGTCTGGATTCAATCACTCCTT**ACCAG**GACCTCCCATAACCAGGGGAGGTTACGTGCTAGGAACACAGGGGAGGGAGGCAGGATGCCCAAGAGTACTCCTCTCTTGTAGGACCT

TGCTCTACTCGTGGCCTTTGCTGAAGGTATCCTCCATGTCTGGATTCAATCACTCCTTACCAGTGTTAAGTTTTTTTAGTAACTAGACGGAAATGAGACCGCCAAGGCAAGCGAGGGTCCAAGAACAGATTTTATTGCAGGCACCCTCGG

TTGGCGTATCCACCATCTTGAGGAGTTTCCCTTCCCACCTGGCCCCAACAACCAGGACCTCCCATAACCAGGGGAGGTTACGTGCTAGGAACACAGGGGAGAGAGGCAGGATGCCAAAGAGTACTCCTCTCT

chr22:45482993-45483143 * chr22:45,483,070 (-)

CCAGTGGAGGGGGAGGTGGGTTGAAGATACTGTTAAACACTTGAATTAACAAAAGTGAATTCCCTGGCAGG**GCCTC**TATATGTTAACTGTATGAGAGCGCCACTCCATGGGCTTCAGGAAGTGACTTAGGCCCTGAGAAGAACAGGCCAC

CCAGTGGAGGGGGAGGTGGGTTGAAGATACTGTTAAACACTTGAATTAACAAAAGTGAATTCCCTGGCAGGGCCTCTGTTAAGTTTTTTTAGTAACTAGACGGAAATGAGACCGCCAAGGC

TCCCTTCCCGCCTGGCCCCAACAGCCTCTATATGTTAACTGTATGAGAGCGCCACTCCATGGGCTTCAGGAAGTGACTTAGG

chr22:57676984-57677134 * chr22:57,677,068 (-)

ATGATTCACCAAGGGTGCTGGGAAGAAGAAAAACTGTGTACATCGAGGCATGCGTGATAGTGAAGAAGTTTCAAAAGAT**ATAAT**GAGACGAGGGAAAAGGGAAGAGAAGAGATTGCGGTCAAGCACTCCGTGCCCTCTAAGAAATGCTGT

ACCAAGGGTGCTGGGAAGAAGAAAAACTGTGTACATCGAGGCATGCGTGATAGTGAAGAAGTTTCAAAAGATATAATTGTTAAGTTTTTTTAGTAACTAGACGGAAATGAGACCGCCAAGGCT

CCCTTCCCGCCTGGCccccaacaATAATGAGACGAGGGAAAAGGGAAGAGAAGAGATTGCGGTCAAGCACTCCGTGCCCTCTAAG

chr26:27059664-27059814 * chr26:27,059,757 (+)

GGAAGCTGAATAAAAGGTACTTGGACTCCATGCACTGTTTTTGCAACTTGCTACGATTATATGATTATTTTTAAATAATTTTTTTAA**ATTAG**TTTTTCAAGAAAATATAATCCAAAATTTGAAATACAACCAAAATGTAATTGTATAATC

ATAAAAGGTACTTGGACTCCATGCACTGTTTTTGCAACTTGCTACGATTATATGATTATTTTTAAATAATTTTTTAAAATTAGTGTTGGGGCCAGGCGGGAAGGGAAACTCCTCAAGATGGCGGATACGCCAAAATGG

GAGGGTGCCTGCAATAAAACCTGTTCTTGGACCCTCGCTTGCCTTGGCGGTCTCATTTCCGTCTAGTTACTAAAAAAAACTTAACAATTAGTTTTTCAAGAAAATATAATCCAAAATTTGAAATACAACCAAAATGTAATTGTATAAT

chr26:28072398-28072548 * chr26:28,072,465 (-)

GGGAGAGGTTTTAAACACATGGATTCCCAGGCTTCATCCTAGTGAACGAGAATCTCTAGGG**AATAG**GGCCTAAGATTGGGATTGTTAGTTTTTCAAAACTCCCTGAGTGGGAATTCTACTGCTGGTGGTGAGGGAGTGACAGGCTGGATT

GTTTTAAACACATGGATTCCCAGGCTTCATCCTAGTGAACGAGAATCTCTAGGGAATAGTGTTAAGTTTTTTTAGTAACTAGACGGAAATGAGACCGCCAAGGCAAGCGAGGGTCCAAGAACAGATTTTATTGCAGGC

CTTCAGCCATTTTGGCGTATCCGCCATCTTGAGGAGTTTCCCTTCCCGCCTGGCCCCAACAAATAGGGCCTAAGATTGGGATTGTTAGTTTTTCAAAACTCCCTGAGTGGGAATTCTACTGCTGGTGGTGAGGGAGTGACAG

chr26:35982371-35982521 Provirus * chr26:35,982,438 (+)

CCTGGGTGGCTCAGTTAAGCATCTGACTCTTGGTTTCAGCTCACATCATGATCTCAGGGTC**CTGAG**ACTGAGCCCCATGGGGAGCTCCATGCTCATTGTGGAGTCTGCTAAAGTTTCTCCCTCTCCCTCTGCCCCTCCCTGTGCTCATTC

CCTGGGTGGCTCAGTTAAGCATCTGACTCTTGGTTTCAGCTCACATCATGATCTCAGGGTCCTGAGTGTTGGGGCCAGGCGGGAAGGGAAACTCCTCAAGATGGCGGATACGCCAAAATGGCTGAAGTTCCTGTCACCACCTCCAC

TCTTGGACCCTCGCTTGCCTTGGCGGTCTCATTTCCGTCTAGTTACTAAAAAAACTTAACACTGAGACTGAGCCCCGTGGGGAGCTCCATGCTCATTGTGGAGTCTGCTAAAGTTTCTCCCTCTCCCTCTGCCCCTCCCTG

chr27: 44066867-44067018 * chr27:44,066,943 (-)

ATACAAGTACTGCCAGCAGTGCATGGCAAGGAGTAAATATTCAATAAACATTAGTTTTTTTTTTAAAGTTTGAATCATTAGCTCTTTGAATCCTGGGCTCTTCTGTAGCTTTCCCTTGGATGCATTGTTTTTTTGTTTTTGTTTTTACAA

GGTGACAGGAACTTCAGCCATTTTGGCGTATCCGCCATCTTGAGGAGTTTCCCTTCCCGCCTGGCCCCAACAATTAGCTCTTTGAATCCTGGGCTCTTCTGTCGCTTTCCCTTGTTTGCATTTTTTTT

chr28:11031827-11031977 * chr28:11,031,931 (+)

GGGGTGATTCCCTGGGCTGTCTTTCTCAGCTCAAAGTGGTTCTCTGTGTACAGTGTTTCTAACTATGGGCTCAGCCCCAAATCTTGGGGCTCTGAAAC**AGTGT**CAGGTGGGAGTTTAGGCTTGGTGTCTTCAAGTTTGGCGCTTGGGTGA

TCCCTGGGCTGTCTTTCTCAGCTCAAAGTGGTTCTCTGTGTACAGTGTTTCTAACTATGGGCTCAGCCCCAAATCTTGGGGCTCTGAAACAGTGTTGTTGGGGCCAGGCGGGAAGGGAAACTCCTCAAGATGGCGGATACGC

GCCCGAGGGTGCCTGCAATAAAATCTGTTCTTGGACCCTCGCTTGCCTTGGCGGTCTCATTTCCGTCTAGTTACTAAAAAAAAACTTAACAAGTGTCAGGTGGGAGTTTAGGCTTGGTGTCTTCAAGTTTGGTGCTTGGGTGA

chr28:19826748-19826898 * chr28:19,826,822 (+)

ATAATCACTCCTTCCCTTGAGTGCTGAGCACAAAAGATGATCTTGTCTCTAAGTTGCCATAAAATGTT**CCTCA**CGTTATTTTTATCTAGGAGTTTTCTATCTCCTCAGTCATAATGCAAGGTACTCAGGTAAAGACCATGGATTTTATTA

ATAATCACTCCTTCCCTTGAGTGCTGAGCACAAAAGATGATCTTGTCTCTAAGTTGCCATAAAATGTTCCTCATGTTGGGGCCAGGCGGGAAGGGAAACTCCTCAAGATGGCGGATACGCCAAAATGGCTGAAGTTCCTGTCACCACC

AATCTGTTCTTGGACCCTCGCTTGCCTTGGCGGTCTCATTTCCGTCTAGTTACTAAAAAAACTTAACACCTCACGTTATTTTTATCTAGGAGTTTTCTATCTCCTCAGTCATAATGCAAGGTACTCAGGTAAAG

chr28:16556145-16556295 * chr28:16,566,219 (+)

CCTTTGGAAACTTCCTTTATCTCTACCCCCTCAGATACAAGTTGGCAATCATCCCCCAAGCATATGAC**CCACC**AATATACATCTGCAGGGTCTCATGACTAAGGTTTTATTGGACAGGAATAAATTACCATTTCCTAACAATAGCTAGCT

CCTTTGGAAACTTCCTTTATCTCTACCCCCTCAGATACAAGTTGGCAATCATCCCCCAAGCATATGACCCACCTGTTGGGGCCAGGCGGGAAGGGAAACTCCTCAAGATGGCGGATACGCCAAAATGGCTGAAGTTCCTGTCA

AATCTGTTCTTGGACCCTCGCTTGCCTTGGCGGGCTCATTTCCGTCTAGTTACTAAAAAAACTTAACACCACCAATATACATCTGCAGGGTCTCATGACTAAGGTTTTATTGGACAGGAATAAATTACCATTTCCTAACAATAG

chr29:30896670-30896820 * chr29:30.896,757 (-)

TCCTATACCTTCGATGGAAAGCCATCTAAGCTTTATATGTAGACAGAAAAATGCGATAAATAACCTTGAATTTTTATAAAG**ATATC**TGTGCAGCATTTTAAAAAATGGAATGGTGTTGGGGAGGGGCAGTGGGGCCTGGAGGAAGTTTAG

CGATGGAAAGCCATCTAAGCTTTATATGTAGACAGAAAAATGCGATAAATAACCTTGAATTTTTATAAAGATATCTGTTAAGTTTTTTTAGTAACTAGACGGAAATGAGACCGCCAAGGCAAGCGAGGGTCCAAGAAC

AGTGGAGGTGGTGACAGGAACTTCAGCCATTTTGGCGTATCCGCCATCTTGAGGAGTTTCCCTTCCCGCCTGGCCCCAACAATATCTGTGCAGCATTTTAAAAAATGGAATGGTGTTGGGGAGGGGCATTGGGGCCTGGAGG

chr30:944428-944578 * chr30:944,498 (+)

GACACAAGAACTTAGAATTATAGGACTTCGACTATATTTGTGATTCATATTGTTTTTTTAAGAA**GTAAA**TTTTAGCAATTCTTTATAAACAGATGATATCTGGGGTACTTAAATATAGAGGTCCAAATGACAGAAAAAGAAACATGAACT

GACACAAGAACTTAGAATTATAGGACTTAGACTATATTTGTGATTCATATTGTTTTTTTAAGAAGTAAATGTTGGGGCCAGGCGGGAAGGGAAACTCCTCAAGATGGCGGATACACCAAAATGGCTGAAGTTCCTGTCACCACCTCCACT

GTTCTTGGACCCTCGCTTGCCTTGGCGGTCTCATTTCCGTCTAGTTACTAAAAAAAACTTAACAGTAAATTTTAGCAATTCTTTATAAACAGATGATATCTGGGGTACTTAAATATAGAGGTCCAAATGACAGAAAAAGAAACATGAACT

chr31:4943770-4943920 * chr31:4,943,865 (+)

AACCAAATTGACAATAGATTGGAAGTTCCAAACACCCCTGCTTCGGGTTTAATTTGCCCAAGTTGCTCCCAGAACTTAGAAACATTTTA**CTTAC**TTGATTACCAGTTTATTATAAAAAGATATAACTCAGAGACAGCCAAATGGAAGAGA

ACCAAATTGACAATAGTTTGGAAGTTCCAAACACCCCTGCTTCGGGTTTAATTTGCCCAAGTTGCTCCCAGAACTTAGAAACATTTTACTTACTGTTGGGGCCAGGCGGGAAGGGAAACTCCTCAAGATGGCGGATACGCCAAAA

GCAATAAAATCTGTTCTTGGACCCTCGCTTGCCTTGGCGGTCTCATTTCCGTCTAGTTACTAAAAAAACTTAACACTTACTTGATTACCAGTTTATTATAAAAAGATATAACTCAGAGACAGCCAAATGGAAG

chr32:7493257-7493407 * chr32:7,493,322 (+)

ATTATGCTTTTATATTCCTTGATTACTGAGAAACTTTGCAAATTATATATATATA----TGTG**TATAT**ATATATACACACACACATATATATATATATATATATATATATATATATTTGTTACTAACAGTTTTAATCTTTGACTATTCCAAT

ATTATGCTTTTATATTCCTTGATTACTGAGAAACTTTGCAAATTTTATATATATATATATGTGTATATTGTTGGGGCCAGGCGGGAAGGGAAACTCCTCAAGATGGCGGATACGCCAAAATGGCTGAAGTTCCTGTCACCACCTCCA

TGTTCTTGGACCCTCGCTTGCCTTGGCGGTCTCATTTCCGTCTAGTTACTAAAAAAACTTAACATATAT-----------------------------------ATATATATATATTTGTTACTAACAGTTTTAATCTTTGACAATTCC

chr32:433663-433813 *chr32:433,745 (-)

GCCAATCCTCCTCACAGGGGGGCAAACACACAATATTATACGGTGGTTGAAACTGCTGCTTAGCCCGGACACCAGTG**CTAAT**ATTTTGTCATTAAAAAACCTTGAAGTCTTGTTTTGATGATCATAGCTCCAGTCTCTTCATCTTCGACT

CTCCTCACAGGGGGGCAAACACACAATATTATACGGTGGTTGAAACTGCTGCTTAGCCCGGACACCAGTGCTAATTGTTAAGTTTTTTTAGTAACTAGACGGAAATGAGACCGCCAAGGCAAGCGAGGGTCCAAGAACAGATT

GAGGTGGTGACAGGAACTTCAGCCATTTTGGCGTATCCGCCATCTTGAGGAGTTTCCCTTCCCGCCTGGCCCCAACACTAATATTTTGTCATTAAAAAACCTTGAAGTCTTGTTTTGATGATCATAGCTCCAGTCTCTTCATCTTC

chr33:22146501-22146651 Provirus * chr32:22,146,581 (-)

AATATTAAGTAGGAAACAAAAGTGCAGAACAGTGTGCATAATATGCTACCTTTGATGAAAGGGAAATAGGAAAGT**ATATG**TGCATGTGTACAAGTGTGTGTGTGTGTGTGTTTGTCTGTGTATCTGTGTGTACATTCCTACCGTTTCACA

AAGTAGGAAACAAAAGTGCAGAACAGTGTGCATAATATGCTACCTTTGATGAAAGGGAAATAGGAAAGTATATGTGTTAAGTTTTTTTAGTAACTAGACGGAAATGAGACCGCCAAGGCAAGCGAGGGTCCAAGAACAGAT

TGGTGACAGGAACTTCAGCCATTTTGGCGTATCCACCATCTTGAGGAGTTTCCCTTCCCGCCTGGCCCCAACAATATGTGCATGTGTACAAGTGTGTGTGTGTGTGT

chr33: 29594983-29595138 * chr33:29,595,068 (+)

GAATAGGTAAATAAAATGTGGTTTAGCCAGATATGTTCGTCTTCTGTGGCTACTGATGTGAATTTTTAGAGTATC**GTTGG**GGTCTGGAGGCAAAGCCAAGAAATAATTATTGAGACATCTTGGGTGCAAAAAGGTGGTTTTATTAAATCA

GAATAGGTAAATAAAATGTGGTTTAGCCAGATATGTTCGTCTTCTGTGGCTACTGATGTGAATTTTTAGAGTATCGTTGGTGTTGGGGCCAGGCGGGAAGGGAAACTCCTCAAGATGGCGGATATGCCAAAATGGCTGAAGTTCCTGTCA

GCAATAAAATCTGTTCCTGGACCCTCGCTTGCCTTGGCGGTCTCATTTCCGTCTAGTTACTAAAAAAACTTAACAGTTGGGGTCTGGAGGCAAAGCCAAGAAATAATTATTGAGACATCTTGGGTGCAAAAAGGTGGTTTTAT

chr33:5338858-5339008 * chr32:5,338,956 (-)

GAAAACCTTTTACACACACACAGGCAAATTAAGTCACTTCCATCTGTTTCTCACACAGTTCTGAATGTATGATTATACCATTGGTTCTATCA**CTCTG**CATTCTAATTATTTGTTTACATGATGCCTCCTCAATACAGTAAGTCACAGCAT

GAAAACCTTTTACACACACACAGGCAAATTAAGTCACTTCCATCTGTTTCTCACACAGTTCTGAATGTATGATTATACCATTGGTTCTATCACTCTGTGTTAAGTTTTTTTAGTAACTAGACGGAAATGAGACCGCCAAGGCAAGCGAG

CTGTCATCCCAAGTGGAGGTGGTGACAGGAACTTCAGCCATTTTGGCGTATCCGCCATCTTGAGGAGTTTCCCTTCCCGCCTGGCCCCAACACTCTGCATTCTAATTATTTGTTTACATGATGCCTCCTCAATACAGTAAGTCACAG

chr34:9822702-9822852 * chr34:9,822,792 (-)

CCATGCTTCTGTCCTCGTCCCCACCAGCTGGCTGTACGGTGTGCCGCTTACAAACCAGCAGACACTTGTAGTGTAAGAGAAGTG**GATTC**CTCCCAGGAATAGTGGCTCCAGGGTGGAGGGAGGCAGAGGCCCTGAGGCAGGAGGATGAGG

CCATGCTTCTGTCCTCGTCCCCACCAGCTGGCTGTACGGTGTGCCGCTTACAAACCAGCAGACACTTGTAGTGTAAGAGAAGTGGATTCTGTTAAGTTTTTTTAGTAACTAGACGGAAATGAGACTGCCAAGGCAAGCGAGGGTCCAAGA

CCAAGTGGAGGTGGTGACAGGAACTTCAGCCATTTTGGCGTATCCGCCATCTTGAGGAGTTTCCCTTCCCGCCTGGCCCCAACAGATTCCTCCCAGGAATAGTGGCTCCAGGGTGGAGGGAGGCAGAGGCCCTGAGGCAGGAGGATGAGG

chr37:29074472-29074622 *chr37:29,074,556 (-)

ATAGTCATACATGCAGCTAATTATACTTAGAATTGCTTGTATTTAAAAATCTCAGTGGATTTCATTTTATTCACTCTC**AGTGG**TACCTTGGTTCTAATCTGTCATAGAAAATTCCTAGAGCTATTCCCCACTCTATCACCAAGATATGGT

ATAGTCATACATGCAGCTAATTATACTTAGAATTGCTTGTATTTAAAAATCTCAGTGGATTTCATTTTATTCACTCTCAGTGGTGTTAAGTTTTTTAGTAACTAGACGGAAATGAGACCGCCAAGGCAAGCGAGGGTCCAAGAA

GACAGGAACTTCAGCCATTTTGGCGTATCCGCCATCTTGAGGAGTTTCCCTTCCCGCCTGGCCCCAACAAGTGGTACCTTGGTTCTAATCTGTCATAGAAAATTCCTAGAGCTATTCCCCACTCTATCACCAAGATATGGT

chr38:17036347-17036497 * chr38:17,036,411 (+)

TATTTTAGGCTTTGTGGGCCATACGGTTTCTGTTAATATTCCACAGAAAACCACCATG**GACAG**TACATAAACAGATGGGCATGGTTGAGTTCCAATAAAACTTTATTTATAAAAGTCAAAAGTGGACTGGGCTGGATTTGGCCTATGAGA

TATTTTAGGCTTTGTGGGCCATACGGTTTCTGTTAATATTCCACAGAAAACCACCATGGACAGTGTTGGGGCCAGGCGGGAAGGGAAACTCCTCAAGATGGCGGATACGCCAAAATGGCTGAAGTTCCTGTCACCACCTCCACTTGGGATG

TGGACCCTCGCTTGCCTTGGCGGTCTCATTTCCGTCTAGTTACTAAAAAAACTTAACAGACAGTACATAAACAGATGGGCATGGTTGAGTTCCAATAAAACTTTATTTATAAAAGTCAAAAGTGGACTGGGCTGGATTTG

chr38:5999944-6000094

The CfERV1z insertion is associated with a gap and present within non-reference structural variation.

Flanking regions are from validations and linked by Sanger sequencing to the chr38:600000 locus; the ‘empty’ flank shown below is not present in the reference.

GAGCTTGTAAATAAACCCTCGTGTGTTTGCGTCGGTGCCGGCTCCTTGGTGGTTTCTCGGATTCGCAATCTTGGGCACAACAGTAAGGGTTTCTTTTTGGGGTGGGGAAAATGTTCTGAAATTAGATGGTGGTGATGGTTACACTGAGAT

TGGTGGCAATGCATAGAGTTCCATATGATGATCATATGGAACTCTAACCTTTTGAAGAATTGCCAGACTGTTCTCCAAAGTGGTAAGTGTGGAGGCCGAGAAAATTAAGGCCATTCCACTTTGAGTTCAGCGTTAGCACAAGTGCAGCCA

chrX:1655486-1655636 *chrX:1,655,533 (-)

ATATACGGCACAGTGGCCTCACCTGGAGAGCTGGGTCCAGT**GTAGG**TACTTTCCCACAGGGTATATTAGCCCCCTGAGCATAATGACAGCAGTGAAGCCATATTTGCGCCTGTCCCCTAGATATGCCCCCCCATGAATTTTTTCATCAGG

ATATACGGCACAGTGGCCTCACCTGGAGGGCTGGGTCCAGTGTAGGTGTTAAGTTTTTTTAGTAACTAGACGGAAATGAGACCGCCAAGGCAAGCGAGGGTCCAAGAACAGATTTTATTGCAGGCACCCTCGGGCGAGGTTCCACG

CCGCCATCTTGAGGAGTTTCCCTTCCCGCCTGGCCCCAACAGTAGGTACTTTCCCACAGGGTATATTAGCCCCCTGAGCATAATGACAGCAGTAAAGCCATATTTGCGCCTGTCCCCTAGATATGCCCCCCCA
