# Supplementary figures and images for: Origin and recent expansion of an endogenous gammaretroviral lineage in domestic and wild canids
Source: Retrovirology. 2019 Mar 7;16:6. doi: 10.1186/s12977-019-0468-z (PMC6407205; doi:10.1186/s12977-019-0468-z)

**Figure S2**

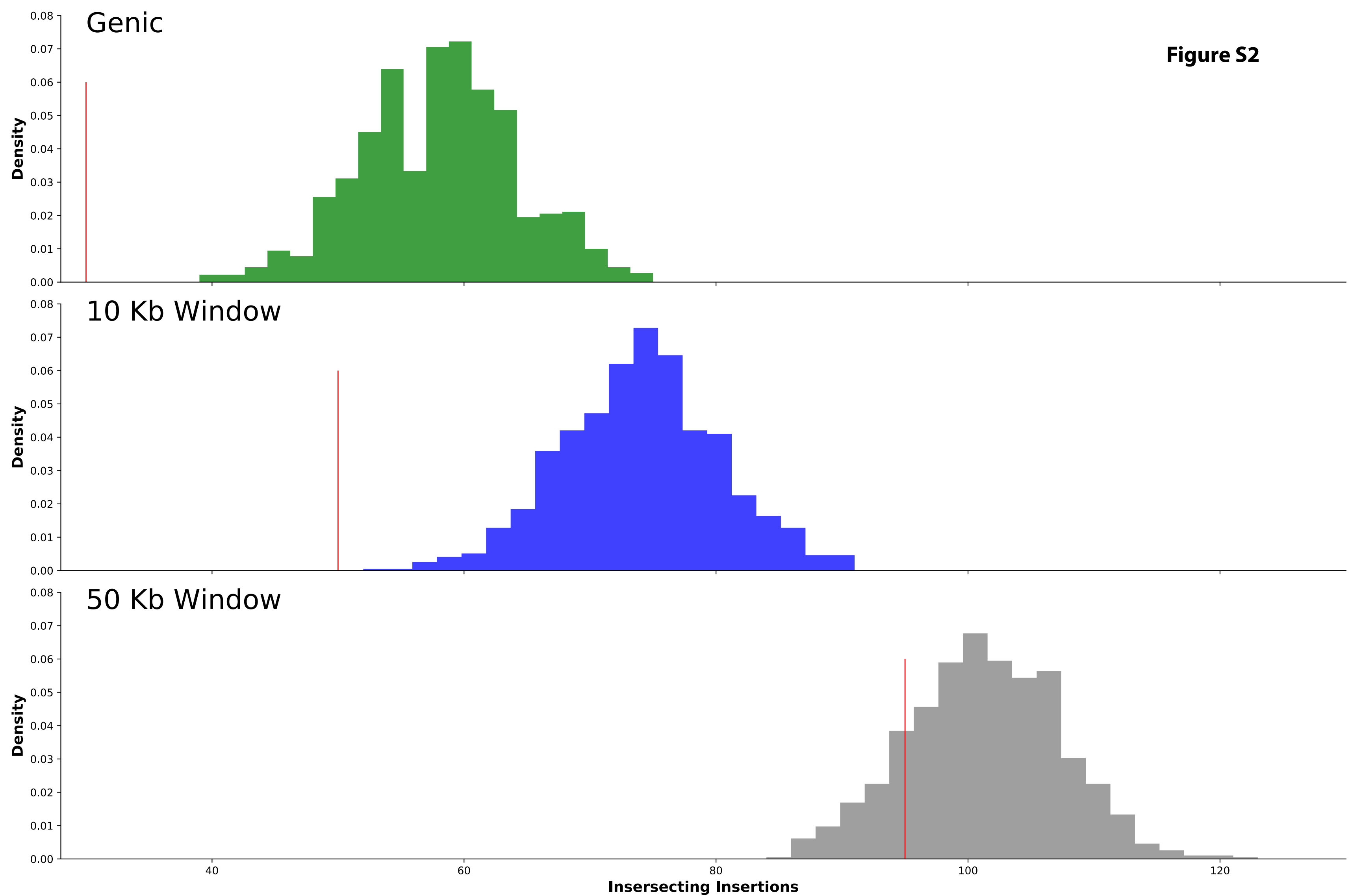

Supplement: Supplementary file 5 — Additional file 5: Figure S2. Depletion of CfERV-Fc1(a) insertions near dog gene models. Following one thousand permutations, the number of gene models that intersect with shuffled CfERV-Fc1(a) insertions are displayed in histograms. Permuted insertions that intersect with at least one Ensembl dog gene model precisely (green), within 10 kb (blue) or 50 kb (gray) are shown. Red lines indicate the observed number of insertions from the true set. [file 12977_2019_468_MOESM5_ESM.pdf]
