# Supplementary material for: Origin and recent expansion of an endogenous gammaretroviral lineage in domestic and wild canids
Source: Retrovirology. 2019 Mar 7;16:6. doi: 10.1186/s12977-019-0468-z (PMC6407205; doi:10.1186/s12977-019-0468-z)
Supplement: Supplementary file 9 — Additional file 9: Figure S3. Annotated CfERV-Fc1(a) consensus provirus. A consensus provirus was deduced from 19 proviruses using BioEdit (http://www.mbio.ncsu.edu/BioEdit/bioedit.html) based on the most commonly represented nucleotide at each site. The consensus nucleotide sequence is shown in black text. The 5′ and 3′ LTRs are labeled with black bars. The translated sequences for the viral genes are indicated below and with bars at the right, with the Gag sequence in blue, Pol in orange, and Env in green. Motifs pertaining to viral functions are labeled appropriately on their translated sequence and general annotated in the right sidebar. Translated start and stop sites are indicated for each of the three genes. Segments for a predicted fusion peptide, membrane-anchoring TM region, and immunosuppressive domain (ISD) were determined using the program Phobius (http://phobius.sbc.su.se). Putative major splice donor and acceptor sites were determined using the program NetGene2 (http://www.cbs.dtu.dk/services/NetGene2/). [file 12977_2019_468_MOESM9_ESM.pdf]

Figure S3

```
                      10        20        30        40        50        60        70        80
CfERV-Fc1 consensus   TGTTGGGGCCAGGCGGGAAGGGAAACTCCTCAAGATGGCGGATACGCCAAAATGGCTGAGGTTCCTGTCACCACCTCCAC

                      90       100       110       120       130       140       150       160
CfERV-Fc1 consensus   TTGGGATGACAGCTTGAGCAGACCCTTACACCTCTCCTTTGGACTTCCTCAACCGAACCCAATGCCCTTCAAACCCCAGA

                     170       180       190       200       210       220       230       240
CfERV-Fc1 consensus   GGAGGAAGTCACCTTTGACTGGTCGAATTGCAATCCTTCCTTTGCATAGTGAGGGTCACTCTGactgGTTGGATTGCAAT

                     250       260       270       280       290       300       310       320
CfERV-Fc1 consensus   CCTTCCTTTGCATATGAGCCAACCAATAGGAAACCGTTCTGCCTTACAACGTTATGTAAACCCCCTACCACCTTGTCTTG

                     330       340       350       360       370       380       390       400
CfERV-Fc1 consensus   GCGCGACTTCCTCGACTCACTCTCTTTCCCCCGTGAGTCGTGGAACCTCGCCCGAGGGTGCCTGCAATAAAATCTGTTCT

                     410       420       430       440       450       460       470       480
CfERV-Fc1 consensus   TGGACCCTCGCTTGCCTTGGCGGTCTCATTTCCGTCTAGTTACTAAAAAAaCTTAACATTTGGTGCCGAAACCCGGGAGG

                     490       500       510       520       530       540       550       560
CfERV-Fc1 consensus   AGATCGAGCCCCTGCAACAGCACGGAGGCTCTCTCCTCTTCCGTCGGACTGGAACTCCGCTCTCTTTCTCTGCTGGGGTC

                     570  SD   580       590       600       610       620       630       640
CfERV-Fc1 consensus   ACCGGACTCCTTGCGGTGAGTGTTCCCTGGTTTCCGACCCTCTCCCGGGGCGCCCTGTCCTAATCGCGGCCGCGTCAGGG

                     650       660       670       680       690       700       710       720
CfERV-Fc1 consensus   CAAACCCTCCTCCGCCACCTGGTGGCTCCGCGGTTCCGGGGAGTAAAGGAGACGTCCTTACTCCACGGTGACCACTTCTG

                     730       740       750       760       770       780       790       800
CfERV-Fc1 consensus   TAAGCAGCAGCTCAATTCACAGTCGAGGGGACGCCCTCCTCCAAGTCTTGAGCTGATACGGGAAGGAGCCATGGGAACCT
                                                                                      M  G  T

                     810       820       830       840       850       860       870       880
CfERV-Fc1 consensus   CCCAGTCCAAATTCGATTCCAAAACGCCTTTAGGATGCCTACTGGCTAATCTCCGAACTCTGGAGTTAGACCAGGACTTA
                      S  Q  S  K  F  D  S  K  T  P  L  G  C  L  L  A  N  L  R  T  L  E  L  D  Q  D  L

                     890       900       910       920       930       940       950       960
CfERV-Fc1 consensus   CGAAGGCGACGCCTTATCCATTACTGTACCGTCGCTTGGCCTCAATATCGGCTGAATAACCAAGCGCAATGGCCACCTGA
                       R  R  R  R  L  I  H  Y  C  T  V  A  W  P  Q  Y  R  L  N  N  Q  A  Q  W  P  P  E

                     970       980       990      1000      1010      1020      1030      1040
CfERV-Fc1 consensus   AGGCACTTTTGATTATCAGATACTTACGGACCTTGATAATCTCTGTAGACGCCAAGGCAAATGGTCTGAGGTGCCCTATG
                        G  T  F  D  Y  Q  I  L  T  D  L  D  N  L  C  R  R  Q  G  K  W  S  E  V  P  Y

                    1050      1060      1070      1080      1090      1100      1110      1120
CfERV-Fc1 consensus   TCCAGGCTTTCTGGACCCTGCGCTCCAGACCAGAACTCTGCTCTAGCTGCTCAACCTTTCAGGTACTCCTAGCCCGCTCT
                      V  Q  A  F  W  T  L  R  S  R  P  E  L  C  S  S  C  S  T  F  Q  V  L  L  A  R  S

                    1130      1140      1150      1160      1170      1180      1190      1200
CfERV-Fc1 consensus   CCCCCGCCGACTCTTCCCCACTCCACCTCTAGAGACTCCAACTTGGCCCCTCCATCCCCCCTCGTGGAGCCTCCTGAAGA
                       P  P  P  T  L  P  H  S  T  S  R  D  S  N  L  A  P  P  S  P  L  V  E  P  P  E  D

                    1210      1220      1230      1240      1250      1260      1270      1280
CfERV-Fc1 consensus   TCTCTCCAGGCCCCCTGTAAGGGCCCCACACCTGTCTCCTCCTCCCTACCAACCCGCTCCCCAACAACCTGTGTCCCCGA
                        L  S  R  P  P  V  R  A  P  H  L  S  P  P  P  Y  Q  P  A  P  Q  Q  P  V  S  P

                    1290      1300      1310      1320      1330      1340      1350      1360
CfERV-Fc1 consensus   CTCCTTCCTCAATCTCCGAAACTCCAGGCCCAGGCCCAGCCTCGAGCATACCCTCTCCAACCGTTCCTCTCCTCCCAGCC
                      T  P  S  S  I  S  E  T  P  G  P  G  P  A  S  S  I  P  S  P  T  V  P  L  L  P  A

                    1370      1380      1390      1400      1410      1420      1430      1440
CfERV-Fc1 consensus   CCAGAACCTCAACCACCTTCCAGCCCCCTCCCTTCTCCTCCTATCTCTGCCCGTACCAGATCCAAAAACTCTTCCCCGGA
                       P  E  P  Q  P  P  S  S  P  L  P  S  P  P  I  S  A  R  T  R  S  K  N  S  S  P  D

                    1450      1460      1470      1480      1490      1500      1510      1520
CfERV-Fc1 consensus   CCTGGTCTGCCCCTTGAGGGAGGTTGCAGGGGCTGAAGGGGTTGTCCGAGTCCATGCGCCCTTTTCTCTACAAGACCTAT
                        L  V  C  P  L  R  E  V  A  G  A  E  G  V  V  R  V  H  A  P  F  S  L  Q  D  L
```

5' LTR (1-457)

PBS

5' UTR

MA

gag

Late Domain

CA

```
                           1530      1540      1550      1560      1570      1580      1590      1600
                   ....|....|....|....|....|....|....|....|....|....|....|....|....|....|....|....|
CfERV-Fc1 consensus CCCAAATAGAAAAACGCTTAGGTTCCTTTTCGGCCAATCCCGACAACTACATCAAGGAATTCCAATACTTGGCGCAGGCC
                    S  Q  I  E  K  R  L  G  S  F  S  A  N  P  D  N  Y  I  K  E  F  Q  Y  L  A  Q  A

                           1610      1620      1630      1640      1650      1660      1670      1680
                   ....|....|....|....|....|....|....|....|....|....|....|....|....|....|....|....|
CfERV-Fc1 consensus TATGACCTAACCTGGCATGACTTACATGTCATCCAGACCACCACCCTCACCACTGAGGAGAGGGAACGTATCCAGGCTGC
                     Y  D  L  T  W  H  K  L  H  V  I  Q  T  T  T  L  T  T  E  E  R  E  R  I  Q  A  A

                           1690      1700      1710      1720      1730      1740      1750      1760
                   ....|....|....|....|....|....|....|....|....|....|....|....|....|....|....|....|
CfERV-Fc1 consensus TGCCCGGGGACACGCTGATCAGGTCCACCTTACCGACGCCACAATGGCGGTCGGTGCTCAGGCGGTCCCCGCCGTAGAGC
                      A  R  G  H  A  D  Q  V  H  L  T  D  A  T  M  A  V  G  A  Q  A  V  P  A  V  E

                           1770      1780      1790      1800      1810      1820      1830      1840
                   ....|....|....|....|....|....|....|....|....|....|....|....|....|....|....|....|
CfERV-Fc1 consensus CAGGCTGGGATTACCAAGACGGTCAAGATGGCCGCCGGCGCCGCGACCACATGGTCCGATGTCTCATCGCTGGCATGCGG
                    P  G  W  D  Y  Q  D  G  Q  D  G  R  R  R  R  D  H  M  V  R  C  L  I  A  G  M  R

                           1850      1860      1870      1880      1890      1900      1910      1920
                   ....|....|....|....|....|....|....|....|....|....|....|....|....|....|....|....|
CfERV-Fc1 consensus GCGGCCTCTAATAAGGCCGTAAATTATGACAAGATCAGAGAAATCATACAGGCCCCTGACGAAAACCCGGCTATATTCCT
                     A  A  S  N  K  A  V  N  Y  D  K  I  R  E  I  I  Q  A  P  D  E  N  P  A  I  F  L

                           1930      1940      1950      1960      1970      1980      1990      2000
                   ....|....|....|....|....|....|....|....|....|....|....|....|....|....|....|....|
CfERV-Fc1 consensus TAACCGGCTGACCGAGGCATTAATTCAGTACACTCGCTTGGACCCGGCCTGTCCCGCGGGGGCCACGGTTCTAGCCACAC
                      N  R  L  T  E  A  L  I  Q  Y  T  R  L  D  P  A  C  P  A  G  A  T  V  L  A  T

                           2010      2020      2030      2040      2050      2060      2070      2080
                   ....|....|....|....|....|....|....|....|....|....|....|....|....|....|....|....|
CfERV-Fc1 consensus ATTTTATCTCTCAGTCAGCCCCAGATATCCGCAAAAAATTAAAGAAAGTCGAGGAAGGCCCTCAGACCCCCATTTCTGAC
                    H  F  I  S  Q  S  A  P  D  I  R  K  K  L  K  K  V  E  E  G  P  Q  T  P  I  S  D

                           2090      2100      2110      2120      2130      2140      2150      2160
                   ....|....|....|....|....|....|....|....|....|....|....|....|....|....|....|....|
CfERV-Fc1 consensus CTAGTGAGAATGGCATTTAAGGTCTTTAACTCCCGTGAGGAAGCCGCAGAGCTGAAGCGACAGGCCAGACTCCAGCAGAA
                     L  V  R  M  A  F  K  V  F  N  S  R  E  E  A  A  E  L  K  R  Q  A  R  L  Q  Q  K

                           2170      2180      2190      2200      2210      2220      2230      2240
                   ....|....|....|....|....|....|....|....|....|....|....|....|....|....|....|....|
CfERV-Fc1 consensus GGTTCAGCTACAAACCCAGGCCTTGGTAGCAGCCCTGCGGCCGGcCGGCTCCAGGAGCCAGCAGAGAGGGGGACCCACCC
                      V  Q  L  Q  T  Q  A  L  V  A  A  L  R  P  A  G  S  R  S  Q  Q  R  G  G  P  T

                           2250      2260      2270      2280      2290      2300      2310      2320
                   ....|....|....|....|....|....|....|....|....|....|....|....|....|....|....|....|
CfERV-Fc1 consensus GCACCCCTCCGGGGGCCTGCTTCAAATGCGGGGCTGAAGGCCATTGGGCCCGTCAGTGCCCCACCCCGAGGGCACCAACT
                    R  T  P  P  G  A  C  F  K  C  G  A  E  G  H  W  A  R  Q  C  P  T  P  R  A  P  T

                           2330      2340      2350      2360      2370      2380      2390      2400
                   ....|....|....|....|....|....|....|....|....|....|....|....|....|....|....|....|
CfERV-Fc1 consensus CGACCATGCCCTCTCTGCCATTTGATGGGCCACTGGAAATCCGACTGCCCTAGCCTCAGGGAATCCTCGGCGCCTCAACG
                     R  P  C  P  L  C  H  L  M  G  H  W  K  S  D  C  P  S  L  R  E  S  S  A  P  Q  R

                           2410      2420      2430      2440      2450      2460      2470      2480
                   ....|....|....|....|....|....|....|....|....|....|....|....|....|....|....|....|
CfERV-Fc1 consensus CGGGGGAAGACCCGAGACGGTGAGTCCAGCCTTCCAACTGCTCGGGCTGGAGGAcGACTGACGGAGCCCAGCCTCGGCCG
                      G  G  R  P  E  T  V  S  P  A  F  Q  L  L  G  L  E  D  D  ▲  R  S  P  A  S  A

                           2490      2500      2510      2520      2530      2540      2550      2560
                   ....|....|....|....|....|....|....|....|....|....|....|....|....|....|....|....|
CfERV-Fc1 consensus CCCCTCTCACCCAGGCCGAGCCCAGGGTCATGCTCCAGGTAGCGGGTAAGTCCATCTCCTTTCTGCTGGATACGGGGGCT
                    A  P  L  T  Q  A  E  P  R  V  M  L  Q  V  A  G  K  S  I  S  F  L  L  D  T  G  A

                           2570      2580      2590      2600      2610      2620      2630      2640
                   ....|....|....|....|....|....|....|....|....|....|....|....|....|....|....|....|
CfERV-Fc1 consensus ACCTATTCTGTTCTGCCTTCCTACGCGGGACCTACTCAACCCTCACCAGTCGCTGTTATGGGGATCGACGGGAACTCCTC
                     T  Y  S  V  L  P  S  Y  A  G  P  T  Q  P  S  P  V  A  V  M  G  I  D  G  N  S  S

                           2650      2660      2670      2680      2690      2700      2710      2720
                   ....|....|....|....|....|....|....|....|....|....|....|....|....|....|....|....|
CfERV-Fc1 consensus CACTCCTAGGGCCACACCTCCCCTTACTTGTAGCCTGGATGGGTTCCCCTTCTCTCACTCATTCCTGGTGATTCCCTCAT
                      T  P  R  A  T  P  P  L  T  C  S  L  D  G  F  P  F  S  H  S  F  L  V  I  P  S

                           2730      2740      2750      2760      2770      2780      2790      2800
                   ....|....|....|....|....|....|....|....|....|....|....|....|....|....|....|....|
CfERV-Fc1 consensus GCCCAGTGCCCCTGCTGGGTAGGGATATCCTCCAAAAGCTAGGGGCAACTATTCATTTGTCCCCCTCTCCTCCCTCCTCA
                    C  P  V  P  L  L  G  R  D  I  L  Q  K  L  G  A  T  I  H  L  S  P  S  P  P  S  S

                           2810      2820      2830      2840      2850      2860      2870      2880
                   ....|....|....|....|....|....|....|....|....|....|....|....|....|....|....|....|
CfERV-Fc1 consensus CCCTCAGCTCGTCTCATCCTATATCTCTCCACTCCATCTACTCCTACCCCGGATCAATTACCCTTCGTGAACCCCCAAGT
                     P  S  A  R  L  I  L  Y  L  S  T  P  S  T  P  T  P  D  Q  L  P  F  V  N  P  Q  V

                           2890      2900      2910      2920      2930      2940      2950      2960
                   ....|....|....|....|....|....|....|....|....|....|....|....|....|....|....|....|
CfERV-Fc1 consensus ATGGGATACCTCGGAACCTGTAGTGGCCTCCCACCACCCTCCCGTCAAAATAAAACTCAAGGACGGTTCCAAGTTCCCCT
                      W  D  T  S  E  P  V  V  A  S  H  H  P  P  V  K  I  K  L  K  D  G  S  K  F  P
```

gag

NC

2x Zinc Finger

pol

PRO

```
                         2970      2980      2990      3000      3010      3020      3030      3040
CfERV-Fc1 consensus  CTAGACCCCAGTTCCCTATCTCCCTCCTCCATCGCCTCGGCCTCAAACCGATCATAGAACGACTAAAGCGTCAGGGACTT
                     S  R  P  Q  F  P  I  S  L  L  H  R  L  G  L  K  P  I  I  E  R  L  K  R  Q  G  L

                         3050      3060      3070      3080      3090      3100      3110      3120
CfERV-Fc1 consensus  CTGATTCCCATAAGCTCTCCCTGTAACTCCCCCATCCTGCCGGTCCGTAAACCCTCCGGAGCTTATCGATTGGTTCAGGA
                      L  I  P  I  S  S  P  C  N  S  P  I  L  P  V  R  K  P  S  G  A  Y  R  L  V  Q  D

                         3130      3140      3150      3160      3170      3180      3190      3200
CfERV-Fc1 consensus  CCTCCGACTCATTAATGAGGCAATAGTCCCTCTCCATCCAGTGGTTCCTAACCCTTATACCCTGCTATCCCATATCCCTC
                       L  R  L  I  N  E  A  I  V  P  L  H  P  V  V  P  N  P  Y  T  L  L  S  H  I  P

                         3210      3220      3230      3240      3250      3260      3270      3280
CfERV-Fc1 consensus  CAAGCACCACTCACTTTACGGTGTTGGACCTGAAGGATGCCTTTTTTACTATTCCCCTACACCCCGATTCCTACTTTCTT
                     P  S  T  T  H  F  T  V  L  D  L  K  D  A  F  F  T  I  P  L  H  P  D  S  Y  F  L

                         3290      3300      3310      3320      3330      3340      3350      3360
CfERV-Fc1 consensus  TTCGCCTTCACCTGGGAAGACCCAGACACACATACCTCTGGACAATTAACCTGGACTGTCCTACCCCAGGGGTTCCGGGA
                      F  A  F  T  W  E  D  P  D  T  H  T  S  G  Q  L  T  W  T  V  L  P  Q  G  F  R  D

                         3370      3380      3390      3400      3410      3420      3430      3440
CfERV-Fc1 consensus  CAGCCCCCATATTTTTGGTCAGGCCTTGGCTGCGGATTTACAACAATGCCTCCTTAAGGCTAGTACCTTACTACAATATG
                       S  P  H  I  F  G  Q  A  L  A  A  D  L  Q  Q  C  L  L  K  A  S  T  L  L  Q  Y

                         3450      3460      3470      3480      3490      3500      3510      3520
CfERV-Fc1 consensus  TAGACGACCTCCTCCTCTGCAGCCCTGCCCTCACCATCTCCCAGGATGACACCACCTCCCTACTTAACTTCCTAGGGAGC
                     V  D  D  L  L  L  C  S  P  A  L  T  I  S  Q  D  D  T  T  S  L  L  N  F  L  G  S

                         3530      3540      3550      3560      3570      3580      3590      3600
CfERV-Fc1 consensus  AAAGGGTATCGGGTCACACCCTCTAAGGCACAACTCTGCACCCCTTCGGTCACTTATCTGGGAATCCACCTGACCCCCAC
                      K  G  Y  R  V  T  P  S  K  A  Q  L  C  T  P  S  V  T  Y  L  G  I  H  L  T  P  T

                         3610      3620      3630      3640      3650      3660      3670      3680
CfERV-Fc1 consensus  CTCTAAATCTCTTACCGGAGACCGTATCCGCCTCCTACGAGAGCTCCAACCCCCTCAGACGGCAGATGAAATACTTTCCT
                       S  K  S  L  T  G  D  R  I  R  L  L  R  E  L  Q  P  P  Q  T  A  D  E  I  L  S

                         3690      3700      3710      3720      3730      3740      3750      3760
CfERV-Fc1 consensus  TCCTCGGGTTGGCTGGCTTCTTTAGACACTGGATTCCAAACTTTTCTATTTTGGCCCGCCCCTTATACCAAGCGGCAAAG
                     F  L  G  L  A  G  F  F  R  H  W  I  P  N  F  S  I  L  A  R  P  L  Y  Q  A  A  K

                         3770      3780      3790      3800      3810      3820      3830      3840
CfERV-Fc1 consensus  GACACTCCCCAGGGTCCTCTCACTGATCCCTCTTCGGTCCGCCGCCTGTTTTCCAAGTTAAGAGACTGTCTCACCGCTGG
                      D  T  P  Q  G  P  L  T  D  P  S  S  V  R  R  L  F  S  K  L  R  D  C  L  T  A  G

                         3850      3860      3870      3880      3890      3900      3910      3920
CfERV-Fc1 consensus  GCCGATACTAACTTTGCCTGACCCTTCAAAACCATTCCACCTTTACACTGATGAGCGGTCGGGTTCAGCTACTGGCCTTT
                       P  I  L  T  L  P  D  P  S  K  P  F  H  L  Y  T  D  E  R  S  G  S  A  T  G  L

                         3930      3940      3950      3960      3970      3980      3990      4000
CfERV-Fc1 consensus  TGGCCCAACCGGTTGGACCTACTTACCGGATTATAGCCTATCTATCTAAGCAGCTAGACAGCACCGCCCGCGGATGGCAG
                     L  A  Q  P  V  G  P  T  Y  R  I  I  A  Y  L  S  K  Q  L  D  S  T  A  R  G  W  Q

                         4010      4020      4030      4040      4050      4060      4070      4080
CfERV-Fc1 consensus  CCCTGCCTTAGGGCGCTCGCAGCTGCTGCCTCCTTAACTAAGGAGGCTCTCAAATTGACTTTGGGACAACCCCTCGTGGT
                      P  C  L  R  A  L  A  A  A  A  S  L  T  K  E  A  L  K  L  T  L  G  Q  P  L  V  V

                         4090      4100      4110      4120      4130      4140      4150      4160
CfERV-Fc1 consensus  TTACTCCCCCCATCGACTTGGGGATCTTCTTAGCCACCGATCCCTGGCCCACCTAACCCCCTCTCGTCTCCAACTCTTCC
                       Y  S  P  H  R  L  G  D  L  L  S  H  R  S  L  A  H  L  T  P  S  R  L  Q  L  F

                         4170      4180      4190      4200      4210      4220      4230      4240
CfERV-Fc1 consensus  ATCTGCTATTCATCGAAAACCCTCAAATCTCCCTCTCTACCTCTCCCCGCTTAAATCCTGCAACTCTGTTGCCTACACCT
                     H  L  L  F  I  E  N  P  Q  I  S  L  S  T  S  P  R  L  N  P  A  T  L  L  P  T  P

                         4250      4260      4270      4280      4290      4300      4310      4320
CfERV-Fc1 consensus  TCGCCTACCTCTGAACCCACTCACTCCTGCCCGCAGCTCATAGAGGATCTCACCCCTCCCCACCCTGGACTCTCTGATCA
                      S  P  T  S  E  P  T  H  S  C  P  Q  L  I  E  D  L  T  P  P  H  P  G  L  S  D  Q

                         4330      4340      4350      4360      4370      4380      4390      4400
CfERV-Fc1 consensus  GCCATTATCCAACCCTGACCGTATACTCTTTGTAGATGGCAGCTCCTTCCTGGCTGCGGATGGTCGGAGACACGCCGCCT
                       P  L  S  N  P  D  R  I  L  F  V  D  G  S  S  F  L  A  A  D  G  R  R  H  A  A

                         4410      4420      4430      4440      4450      4460      4470      4480
CfERV-Fc1 consensus  ATGCTGTAGTCACCCCAGAGACGGTAGTGGAGACAGTCCCCCTCCCAATTGGGACTACTTCCCAAAGGGCTGAACTTATA
                     Y  A  V  V  T  P  E  T  V  V  E  T  V  P  L  P  I  G  T  T  S  Q  R  A  E  L  I
```

RT

RT Active Site

pol

RNase H

DEDD RNA hydrolysis

```
                    4490      4500      4510      4520      4530      4540      4550      4560
CfERV-Fc1 consensus GCTCTCACCAGGGCTCTACATCTATCTAAGGGACAACGAGTCACCATCTACACCGACTCAAAATATGCCTATCTCATCGT
                     A  L  T  R  A  L  H  L  S  K  G  Q  R  V  T  I  Y  T  D  S  K  Y  A  Y  L  I  V

                    4570      4580      4590      4600      4610      4620      4630      4640
CfERV-Fc1 consensus TCATACTCATTCCGTCCTCTGGCAGGAGCGGGGATTTTTAACCACCAAGGGGACGCCTATAGTAAATGGACCTCTCATTG
                      H  T  H  S  V  L  W  Q  E  R  G  F  L  T  T  K  G  T  P  I  V  N  G  P  L  I

                    4650      4660      4670      4680      4690      4700      4710      4720
CfERV-Fc1 consensus CCAAATTGCTTGAGGCCCTTAGTCTGCCCACTGAGGTTGCAATCGTTCACTGTAGGGGCCATCAGACTTCTAAAGACATG
                    A  K  L  L  E  A  L  S  L  P  T  E  V  A  I  V  H  C  R  G  H  Q  T  S  K  D  M

                    4730      4740      4750      4760      4770      4780      4790      4800
CfERV-Fc1 consensus GTCTCCATAGGAAATAATAAGGCCGACTCAGTGGCCAGGGAAACGGCCTTAAGTAACCCAATATCTCCCATCCTCTTCCT
                     V  S  I  G  N  N  K  A  D  S  V  A  R  E  T  A  L  S  N  P  I  S  P  I  L  F  L

                    4810      4820      4830      4840      4850      4860      4870      4880
CfERV-Fc1 consensus TAATACCCCTCATCGACCTTTCTACTCTATAAAGGAAACTCAAGCCCTCCAGGCCCTGGGAGGAAAGGCAGAAAGTAAAG
                      N  T  P  H  R  P  F  Y  S  I  K  E  T  Q  A  L  Q  A  L  G  G  K  A  E  S  K

                    4890      4900      4910      4920      4930      4940      4950      4960
CfERV-Fc1 consensus GATGGATTTACATCCGAGGGAAGATTGCCCTCCCAGAAAACCTGGCCCATACCCTAATTACTGATATCCACCAATCTCTC
                    G  W  I  Y  I  R  G  K  I  A  L  P  E  N  L  A  H  T  L  I  T  D  I  H  Q  S  L

                    4970      4980      4990      5000      5010      5020      5030      5040
CfERV-Fc1 consensus CATATTGGCCCAAGGGCACTGAACCAGTTTCTCCAGCCCCTGTTTTACTATCCATCCCTACCTAAGGTGATTGAGGCTGT
                     H  I  G  P  R  A  L  N  Q  F  L  Q  P  L  F  Y  Y  P  S  L  P  K  V  I  E  A  V

                    5050      5060      5070      5080      5090      5100      5110      5120
CfERV-Fc1 consensus CCATAGGGCTTGTAAAACCTGCTCGGCCGTAAATGCACAGGGAGGAATCCGCAGGCCGGGGCCTAACCATCAGCTCCGAG
                      H  R  A  C  K  T  C  S  A  V  N  A  Q  G  G  I  R  R  P  G  P  N  H  Q  L  R

                    5130      5140      5150      5160      5170      5180      5190      5200
CfERV-Fc1 consensus GCCATCAGCCCGGTGAAGACTGGCAGCTGGACTTCACTCACATGCCCCGCCATAAAGCCTTTCGTTATCTACTGACTTTG
                    G  H  Q  P  G  E  D  W  Q  L  D  F  T  H  M  P  R  H  K  A  F  R  Y  L  L  T  L

                    5210  SA  5220      5230      5240      5250      5260      5270      5280
CfERV-Fc1 consensus GTTGATACTTTTACAGGATGGATTGAGGCATACCCCACAGCCAGAGAGACTGCAGATGTGGTGGCCACAATCCTCATCGA
                     V  D  T  F  T  G  W  I  E  A  Y  P  T  A  R  E  T  A  D  V  V  A  T  I  L  I  E

                    5290      5300      5310      5320      5330      5340      5350      5360
CfERV-Fc1 consensus GCACATCATCCCGAGGTTTGGGTTACCCCGGACCCTACAGTCAGACAACGGGCCGGCATTTATCTCCAGTGTGACCCAAC
                      H  I  I  P  R  F  G  L  P  R  T  L  Q  S  D  N  G  P  A  F  I  S  S  V  T  Q

                    5370      5380      5390      5400      5410      5420      5430      5440
CfERV-Fc1 consensus AGGTGGCCGAGAGCCTCAACATTACCTGGAAGCTGCACATCCCCTACCACCCTCAGTCTTCGGGTAAGGTGGAAAGGGCC
                    Q  V  A  E  S  L  N  I  T  W  K  L  H  I  P  Y  H  P  Q  S  S  G  K  V  E  R  A

                    5450      5460      5470      5480      5490      5500      5510      5520
CfERV-Fc1 consensus AACGGGCTACTTAAGGCTCAACTCACTAAACTTACCCTGGAGACTCGCCTGTCGTGGCCCACACTGTTACCTATAGCTCT
                     N  G  L  L  K  A  Q  L  T  K  L  T  L  E  T  R  L  S  W  P  T  L  L  P  I  A  L

                    5530      5540      5550      5560      5570      5580      5590      5600
CfERV-Fc1 consensus CACCAGACTCCGGGCgTCCCCCCGAGGACCATCAGGTTTGAGTCCCTTTGAGTTACTGTATGGTCGGCCCTTCCTTATCA
                      T  R  L  R  A  S  P  R  G  P  S  G  L  S  P  F  E  L  L  Y  G  R  P  F  L  I

                    5610      5620      5630      5640      5650      5660      5670      5680
CfERV-Fc1 consensus ACCACAACCTCCCGGCCATTCCTCCACCTCTTTTATCCTATCTGCCTTACCTTACCCTCCTCCGGGCCCTCTTGAGAGCC
                    N  H  N  L  P  A  I  P  P  P  L  L  S  Y  L  P  Y  L  T  L  L  R  A  L  L  R  A

                    5690      5700      5710      5720      5730      5740      5750      5760
CfERV-Fc1 consensus CATGCTGATGCTGTCATTCCGGCCCCGACCGACAATGCCTCCGAGGAAGCCTCTCCACGAGACTTATCCCCAGGGGACCA
                     H  A  D  A  V  I  P  A  P  T  D  N  A  S  E  E  A  S  P  R  D  L  S  P  G  D  Q
                      M  L  M  L  S  F  R  P  R  P  T  M  P  P  R  K  P  L  H  E  T  Y  P  Q  G  T

                    5770      5780      5790      5800      5810      5820      5830      5840
CfERV-Fc1 consensus GGTCCTTCTCAGAAATCTGCAGCCAGGCTCCCTGCAGACCCGATGGACCGGACCTCACACGGTCATCCTCACCACCCCAA
                      V  L  L  R  N  L  Q  P  G  S  L  Q  T  R  W  T  G  P  H  T  V  I  L  T  T  P
                    R  S  F  S  E  I  C  S  Q  A  P  C  R  P  D  G  P  D  L  T  R  S  S  S  P  P  Q

                    5850      5860      5870      5880      5890      5900      5910      5920
CfERV-Fc1 consensus CCGCGGCAAAATTGCTGGGACATACGGCATGGGTGCACATCAACAACCTTAAACGGGCACCCACAGGTATCGAATGGACC
                    T  A  A  K  L  L  G  H  T  A  W  V  H  I  N  N  L  K  R  A  P  T  G  I  E  W  T
                     P  R  Q  N  C  W  D  I  R  H  G  C  T  S  T  T  L  N  G  H  P  Q  V  S  N  G  P

                    5930      5940      5950      5960      5970      5980      5990      6000
CfERV-Fc1 consensus TCCCAGATGGTGGGACCCACCAAACTCCGCCTGGCTAGGGCTCCTTCTCATACTTCTCCTGAGCCCCCCGATCCAAGCGG
                     S  Q  M  V  G  P  T  K  L  R  L  A  R  A  P  S  H  T  S  P  E  P  P  D  P  S  G
                      P  R  W  W  D  P  P  N  S  A  W  L  G  L  L  L  I  L  L  L  S  P  P  I  Q  A
```

RNase H

H_2C_2 DNA Binding

pol

IN

$DDX_{35}E$ protease resistant core

env

SU

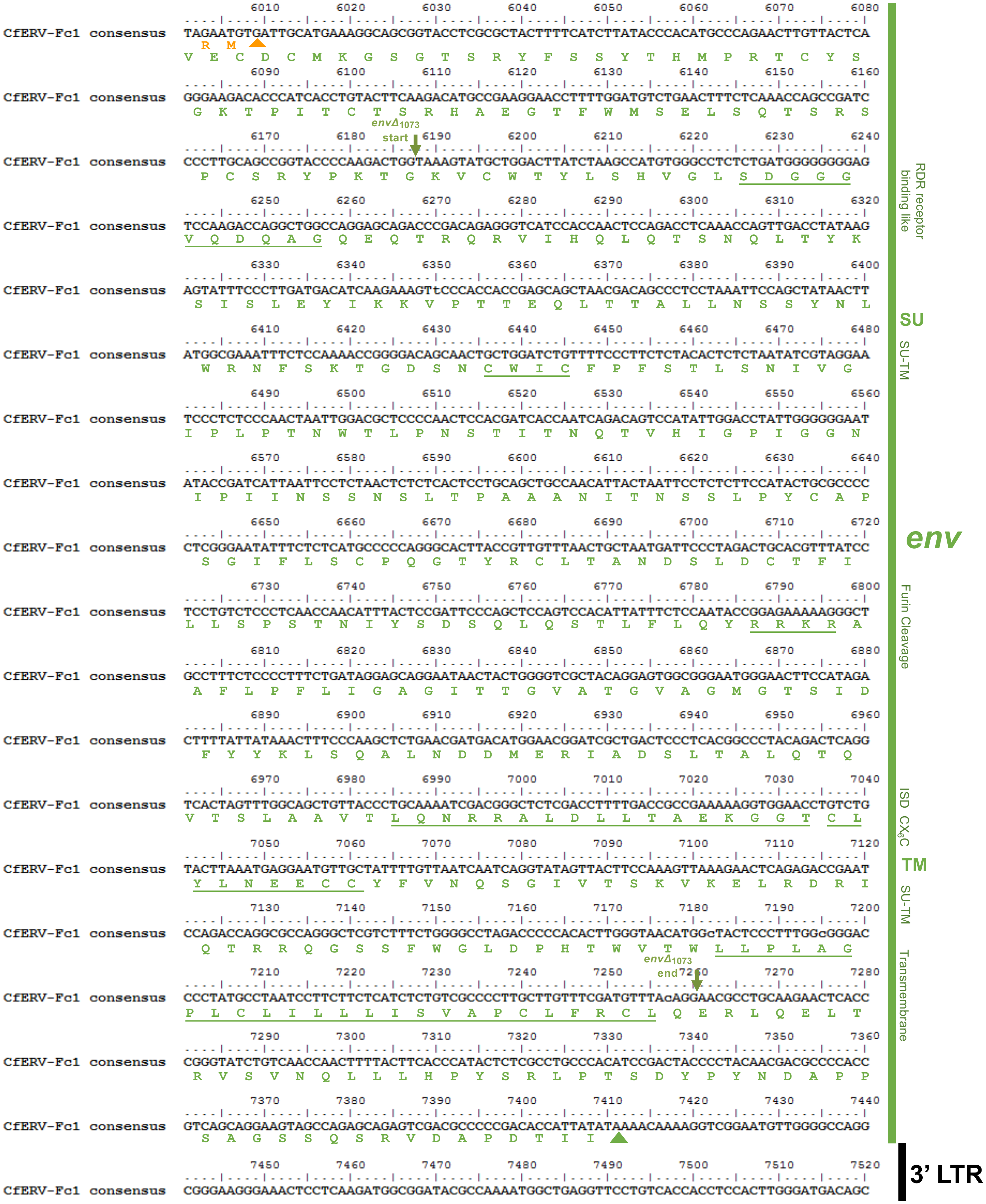

6010 6020 6030 6040 6050 6060 6070 6080
CfERV-Fc1 consensus TAGAATGTGATTGCATGAAAGGCAGCGGTACCTCGCGCTACTTTTCATCTTATACCCACATGCCCAGAACTTGTTACTCA
R M
V E C D C M K G S G T S R Y F S S Y T H M P R T C Y S
6090 6100 6110 6120 6130 6140 6150 6160
CfERV-Fc1 consensus GGGAAGACACCCATCACCTGTACTTCAAGACATGCCGAAGGAACCTTTTGGATGTCTGAACTTTCTCAAACCAGCCGATC
G K T P I T C T S R H A E G T F W M S E L S Q T S R S
envΔ1073 start
6170 6180 6190 6200 6210 6220 6230 6240
CfERV-Fc1 consensus CCCTTGCAGCCGGTACCCCAAGACTGGTAAAGTATGCTGGACTTATCTAAGCCATGTGGGCCTCTCTGATGGGGGGGGAG
P C S R Y P K T G K V C W T Y L S H V G L S D G G G
6250 6260 6270 6280 6290 6300 6310 6320
CfERV-Fc1 consensus TCCAAGACCAGGCTGGCCAGGAGCAGACCCGACAGAGGGTCATCCACCAACTCCAGACCTCAAACCAGTTGACCTATAAG
V Q D Q A G Q E Q T R Q R V I H Q L Q T S N Q L T Y K
6330 6340 6350 6360 6370 6380 6390 6400
CfERV-Fc1 consensus AGTATTTCCCTTGATGACATCAAGAAAGTtCCCACCACCGAGCAGCTAACGACAGCCCTCCTAAATTCCAGCTATAACTT
S I S L E Y I K K V P T T E Q L T T A L L N S S Y N L
6410 6420 6430 6440 6450 6460 6470 6480
CfERV-Fc1 consensus ATGGCGAAATTTCTCCAAAACCGGGGACAGCAACTGCTGGATCTGTTTTCCCTTCTCTACACTCTCTAATATCGTAGGAA
W R N F S K T G D S N C W I C F P F S T L S N I V G
6490 6500 6510 6520 6530 6540 6550 6560
CfERV-Fc1 consensus TCCCTCTCCCAACTAATTGGACGCTCCCCAACTCCACGATCACCAATCAGACAGTCCATATTGGACCTATTGGGGGGAAT
I P L P T N W T L P N S T I T N Q T V H I G P I G G N
6570 6580 6590 6600 6610 6620 6630 6640
CfERV-Fc1 consensus ATACCGATCATTAATTCCTCTAACTCTCTCACTCCTGCAGCTGCCAACATTACTAATTCCTCTCTTCCATACTGCGCCCC
I P I I N S S N S L T P A A A N I T N S S L P Y C A P
6650 6660 6670 6680 6690 6700 6710 6720
CfERV-Fc1 consensus CTCGGGAATATTTCTCTCATGCCCCCAGGGCACTTACCGTTGTTTAACTGCTAATGATTCCCTAGACTGCACGTTTATCC
S G I F L S C P Q G T Y R C L T A N D S L D C T F I
6730 6740 6750 6760 6770 6780 6790 6800
CfERV-Fc1 consensus TCCTGTCTCCCTCAACCAACATTTACTCCGATTCCCAGCTCCAGTCCACATTATTTCTCCAATACCGGAGAAAAAGGGCT
L L S P S T N I Y S D S Q L Q S T L F L Q Y R R K R A
6810 6820 6830 6840 6850 6860 6870 6880
CfERV-Fc1 consensus GCCTTTCTCCCCTTTCTGATAGGAGCAGGAATAACTACTGGGGTCGCTACAGGAGTGGCGGGAATGGGAACTTCCATAGA
A F L P F L I G A G I T T G V A T G V A G M G T S I D
6890 6900 6910 6920 6930 6940 6950 6960
CfERV-Fc1 consensus CTTTTATTATAAACTTTCCCAAGCTCTGAACGATGACATGGAACGGATCGCTGACTCCCTCACGGCCCTACAGACTCAGG
F Y Y K L S Q A L N D D M E R I A D S L T A L Q T Q
6970 6980 6990 7000 7010 7020 7030 7040
CfERV-Fc1 consensus TCACTAGTTTGGCAGCTGTTACCCTGCAAAATCGACGGGCTCTCGACCTTTTGACCGCCGAAAAAGGTGGAACCTGTCTG
V T S L A A V T L Q N R R A L D L L T A E K G G T C L
7050 7060 7070 7080 7090 7100 7110 7120
CfERV-Fc1 consensus TACTTAAATGAGGAATGTTGCTATTTTGTTAATCAATCAGGTATAGTTACTTCCAAAGTTAAAGAACTCAGAGACCGAAT
Y L N E E C C Y F V N Q S G I V T S K V K E L R D R I
7130 7140 7150 7160 7170 7180 7190 7200
CfERV-Fc1 consensus CCAGACCAGGCGCCAGGGCTCGTCTTTCTGGGGCCTAGACCCCCACACTTGGGTAACATGGcTACTCCCTTTGGcGGGAC
Q T R R Q G S S F W G L D P H T W V T W L L P L A G
envΔ1073 end
7210 7220 7230 7240 7250 7260 7270 7280
CfERV-Fc1 consensus CCCTATGCCTAATCCTTCTTCTCATCTCTGTCGCCCCTTGCTTGTTTCGATGTTTAcAGGAACGCCTGCAAGAACTCACC
P L C L I L L L I S V A P C L F R C L Q E R L Q E L T
7290 7300 7310 7320 7330 7340 7350 7360
CfERV-Fc1 consensus CGGGTATCTGTCAACCAACTTTTACTTCACCCATACTCTCGCCTGCCCACATCCGACTACCCCTACAACGACGCCCCACC
R V S V N Q L L L H P Y S R L P T S D Y P Y N D A P P
7370 7380 7390 7400 7410 7420 7430 7440
CfERV-Fc1 consensus GTCAGCAGGAAGTAGCCAGAGCAGAGTCGACGCCCCCGACACCATTATATAAAACACAAAAGGTCGGAATGTTGGGGCCAGG
S A G S S Q S R V D A P D T I I
7450 7460 7470 7480 7490 7500 7510 7520
CfERV-Fc1 consensus CGGGAAGGGAAACTCCTCAAGATGGCGGATACGCCAAAATGGCTGAGGTTCCTGTCACCACCTCCACTTGGGATGACAGC
RDR receptor binding like
SU
SU-TM
env
Furin Cleavage
ISD CX6C
TM
SU-TM
Transmembrane
3' LTR

```
                            7530      7540      7550      7560      7570      7580      7590      7600
                    ....|....|....|....|....|....|....|....|....|....|....|....|....|....|....|....|
CfERV-Fc1 consensus TTGAGCAGACCCTTACACCTCTCCTTTGGACTTCCTCAACCGAACCCAATGCCCTTCAAACCCCAGAGGAGGAAGTCACC

                            7610      7620      7630      7640      7650      7660      7670      7680
                    ....|....|....|....|....|....|....|....|....|....|....|....|....|....|....|....|
CfERV-Fc1 consensus TTTGACTGGTCGAATTGCAATCCTTCCTTTGCATAGTGAGGGTCACTCTGACTGGTTGGATTGCAATCCTTCCTTTGCAT

                            7690      7700      7710      7720      7730      7740      7750      7760
                    ....|....|....|....|....|....|....|....|....|....|....|....|....|....|....|....|
CfERV-Fc1 consensus ATGAGCCAACCAATAGGAAACCGTTCTGCCTTACAACGTTATGTAAACCCCCTACCACCTTGTCTTGGCGCGACTTCCTC

                            7770      7780      7790      7800      7810      7820      7830      7840
                    ....|....|....|....|....|....|....|....|....|....|....|....|....|....|....|....|
CfERV-Fc1 consensus GACTCACTCTCTTTCCCCCGTGAGTCGTGGAACCTCGCCCGAGGGTGCCTGCAATAAAATCTGTTCTTGGACCCTCGCTT

                            7850      7860      7870      7880
                    ....|....|....|....|....|....|....|....|.....|
CfERV-Fc1 consensus GCCTTGGCGGTCTCATTTCCGTCTAGTTACTAAAAAAACTTAACA
```

3' LTR
(7428-7885)
